# Single‐Cell Transcriptome Atlas and Regulatory Dynamics in Developing Cotton Anthers

**DOI:** 10.1002/advs.202304017

**Published:** 2023-11-17

**Authors:** Yanlong Li, Huanhuan Ma, Yuanlong Wu, Yizan Ma, Jing Yang, Yawei Li, Dandan Yue, Rui Zhang, Jie Kong, Keith Lindsey, Xianlong Zhang, Ling Min

**Affiliations:** ^1^ National Key Laboratory of Crop Genetic Improvement & Hubei Hongshan Laboratory Huazhong Agricultural University Wuhan Hubei 430070 China; ^2^ Department of Biosciences Durham University Durham 27710 UK; ^3^ Institute of Economic Crops Xinjiang Academy of Agricultural Sciences Xinjiang 830091 China

**Keywords:** anthers, high temperature, single‐cell multi‐omics, single‐cell RNA sequencing (scRNA‐seq), tapetum

## Abstract

Plant anthers are composed of different specialized cell types with distinct roles in plant reproduction. High temperature (HT) stress causes male sterility, resulting in crop yield reduction. However, the spatial expression atlas and regulatory dynamics during anther development and in response to HT remain largely unknown. Here, the first single‐cell transcriptome atlas and chromatin accessibility survey in cotton anther are established, depicting the specific expression and epigenetic landscape of each type of cell in anthers. The reconstruction of meiotic cells, tapetal cells, and middle layer cell developmental trajectories not only identifies novel expressed genes, but also elucidates the precise degradation period of middle layer and reveals a rapid function transition of tapetal cells during the tetrad stage. By applying HT, heterogeneity in HT response is shown among cells of anthers, with tapetal cells responsible for pollen wall synthesis are most sensitive to HT. Specifically, HT shuts down the chromatin accessibility of genes specifically expressed in the tapetal cells responsible for pollen wall synthesis, such as *QUARTET 3* (*QRT3*) and *CYTOCHROME P450 703A2* (*CYP703A2*), resulting in a silent expression of these genes, ultimately leading to abnormal pollen wall and male sterility. Collectively, this study provides substantial information on anthers and provides clues for heat‐tolerant crop creation.

## Graphical abstract

Anther development and function have central role in plant reproduction. Here, the first exploration of the spatial expression profiles and regulatory dynamics of different cell types during cotton anther development and in response to high temperature open a new chapter in plant anther research and provides crucial clue for breeding heat‐tolerant crops.

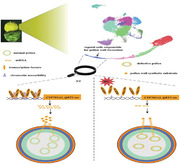

## 1 Introduction

The anther is essential for the production of male gametes in angiosperms and consists of seven tissues: epidermis, endothecium, middle layer, tapetum, microspores, connective tissue, and vascular tissue.^[^ ^1^ ^]^ Anther development is a complex process, divided into 14 consecutive stages in *Arabidopsis*, rice, and cotton.^[^ ^2^, ^3^, ^4^ ^]^ Some genes regulating anther development have been identified, and loss of function of these genes can lead to male sterility.^[^ ^1^, ^5^ ^]^ However, most previous studies have been based on individual or a few genes, while the gene regulatory network has not been explored. Therefore, an in‐depth analysis of regulatory mechanisms is required for a fuller understanding of anther developmental processes and crop breeding applications. The ultimate function of anther development is to provide healthy and mature pollen grains. The pollen wall has many biological functions, such as protection of male gametes, assisting pollen dispersal, and ensuring correct identification between pollen and stigma.^[^ ^6^ ^]^ The process of pollen wall synthesis is a complex and sophisticated biological process that is regulated by a large number of genes, which are well understood in *Arabidopsis* and rice.^[^ ^7^ ^]^ The tapetum, which is the layer of cells most closely associated physically with the microspores, plays an essential role in the correct formation of the pollen wall.^[^ ^8^ ^]^ However, how the formation of the pollen wall is finely regulated by the tapetum at the single‐cell level require further analysis.

With global warming, extreme high temperatures (HTs) are occurring with increasing frequency.^[^ ^9^ ^]^ HT stress often causes significant damage to the growth and development of various crops, resulting in 2−18% crop yield loss while the average annual temperatures in crop cultivated regions have risen by 1 °C.^[^ ^10^, ^11^ ^]^ Cotton, an important summer cash crop, often encounters HT stress during the flowering period, resulting in anther sterility and severe yield loss.^[^ ^12^, ^13^ ^]^ Although HT disrupts physiological processes within cotton anthers, including starch synthesis,^[^ ^14^ ^]^ auxin metabolism,^[^ ^15^ ^]^ jasmonic acid (JA) metabolism,^[^ ^16^ ^]^ these studies have mainly focused on revealing the metabolic basis of male sterility under HT, while the specific mechanism of response to HT stress during anther development at the single‐cell level is not known.

Cells are the basic structural and functional units of an organism, but they are interdependent and interact with each other to form a hierarchically ordered system.^[^ ^17^ ^]^ Traditional studies often analyze whole tissue and obtain the average of all cellular information of the whole tissue, which obscures the nature of cellular heterogeneity. Single‐cell transcriptome sequencing (scRNA‐seq) has begun to make its mark in plant research by exploring developmental differentiation and abiotic stress responses of different cell types. Single‐cell transcriptional profiles have been constructed of tissues such as root tips of rice,^[^ ^18^ ^]^ stems of poplar,^[^ ^19^ ^]^ ovules and cotyledons of cotton.^[^ ^20^, ^21^ ^]^ In *Arabidopsis*, researchers have resolved the molecular mechanism of root epidermal cell proliferation induced by low phosphorus stress through single‐cell transcriptome.^[^ ^22^ ^]^ However, in‐depth single‐cell sequencing of anther tissues in plants and construction of a single‐cell atlas of cotton anthers under HT have not been performed. To understand the single‐cell transcriptome atlas and regulatory dynamics in response to HT stress in the cotton anther, we performed single‐nuclei RNA sequencing (snRNA‐seq) and single‐nuclei multi‐omics under normal temperature (NT) and HT, exploring gene expression patterns and chromatin accessibility. Our study provides the first transcriptional information of the different cell types and the developmental dynamics of meiotic cells, middle layer cells, and tapetal cells in cotton tetrad anthers. We show the degradation of the middle layer ends at stage 11 in cotton anthers, which is different with other plant anthers. We also found a unique HT response of each cell type in the cotton anther, whereby the tapetal cells disappear in response to HT. These results provide an unique perspective on the response of cotton anthers to HT stress at the single cell level.

## 2 Results

### 2.1 Construction of Single‐Cell Transcriptome Atlas of the Developing Cotton Anther

**Figure 1:**
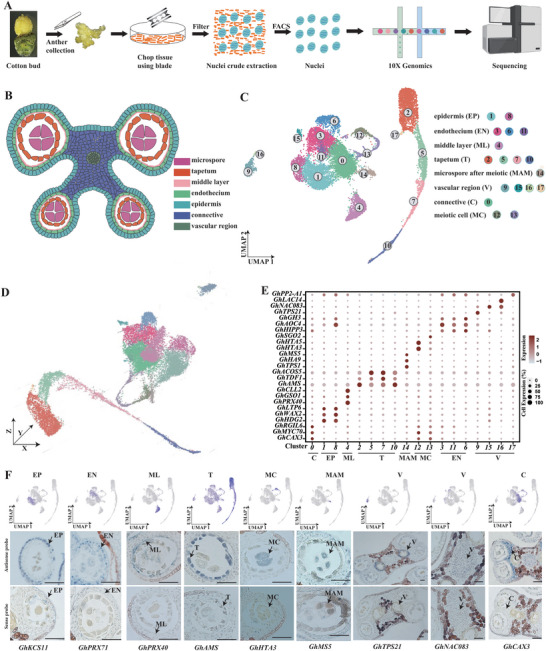
Generation of a cotton anther cell expression atlas. A) Schematic of single‐cell sequencing in cotton anthers. B) Sketches of anatomy of cotton anther. C) An integrated uniform manifold approximation and projection (UMAP) visualization of 18 cell clusters in cotton anthers. Each dot denotes a single‐cell. Colors denote corresponding cell clusters. D) 18 cell clusters displayed by 3D UMAP scatterplots. Cluster names and colors are the same as in (C). E) Expression patterns of representative cluster‐specific marker genes on UMAP. Dot diameter indicates the proportion of cluster cells expressing a given gene. F) The expression patterns of selected marker genes in cotton anthers using UMAP plots and RNA in situ hybridization assays. *KCS11*, *3‐KETOACYL‐COA SYNTHASE 11*; *PRX71*, *PEROXIDASE 71*; *PRX40*, *PEROXIDASE40*; *AMS*, *ABORTED MICROSPORES*; *HTA3*, *HISTONE H2A 3*; *MS5*, *MALE‐STERILE 5*; *TPS21*, *TERPENE SYNTHASE 21*; *NAC083*, *NAC DOMAIN CONTAINING PROTEIN 83*; *CAX3*, *CATION EXCHANGER 3*; EP, epidermis; EN, endothecium; ML, middle layer; T, tapetum; MAM, microspore after meiotic; V, vascular region; C, connective; MC, meiotic cell.

To generate a single‐cell atlas from developing cotton anthers, we used 6–7 mm cotton buds, a stage with major developmental and architectural decisions, including completion of meiosis, formation of tetrads, and the initiation of pollen wall synthesis.^[^ ^3^ ^]^ To ensure the accuracy of the sample period, we microscopically examined 3–5 anthers in each bud, and if the tetrads were observed in these anthers, the remained anthers from the same bud were retained (**Figure** **1**A). Because previous studies have shown that the process of protoplast isolation has artefactual effects and that the affected genes vary considerably from batch to batch, which may affect the clustering of cells and the annotation of cell types,^[^ ^18^, ^23^ ^]^ we isolated nuclei and subjected them to snRNA‐seq assay using a commercial 10Χ chromium platform (Figure 1A). We profiled 25050 individual cells and used standard computational pipelines (Cell Ranger provided by 10Χ Genomics) to align the raw sequencing data to the cotton genome (Table S1, Supporting Information). After data filtration at both the cell and gene levels, we derived a gene expression matrix of 53755 genes (76% of the total annotated genes in the *Gossypium hirsutum* genome) across 17732 filtered cells (Table S1, Supporting Information).

Based on this gene expression matrix, we performed principal compone**n**t analysis (PCA) across 2000 genes showing expression variation and identified 100 statistically significant principal components (PCs). These PCs were used to build a k‐nearest neighbor graph of the anther cells, which was then partitioned into different numbers of clusters at different resolutions (0, 0.2, 0.4, 0.6, 0.8, 1.0) (Figure S1A, Supporting Information). Considering the known distinguishing anther cell types and developmental events during this period (Figure 1B), we chose a resolution of 0.6 for further analysis. At this resolution, the cells were classified into 18 clusters and were visualized by t‐distributed stochastic neighborhood embedding (t‐SNE; Figure S1B, Supporting Information) and uniform manifold approximation and projection (UMAP; Figure 1C), with the UMAP being more meaningful for the organization of cell clusters.3D UMAP plots were constructed to determine the spatial and connective distribution of cell clusters (Figure 1D; Movie S1, Supporting Information). We extracted the expression data of the top 10 marker genes from each cluster in all cells for heatmap analysis and found that these marker genes were enriched in the cluster they represented (Figure S2, Supporting Information). This allowed us to establish by snRNA‐seq a tetrad stage anther cell atlas with 18 cell clusters with distinct expression characteristics.

### 2.2 Identification of Cell Types in the Cotton Anthers

To annotate cell clusters in the cotton anther cell atlas faithfully, we first identified a series of cluster‐enriched genes and cluster‐specific marker genes by analyzing differentially expressed genes (DEGs) among the clusters (Figure 1E; Table S2, Supporting Information). We then predicted identities for each meta‐cluster by finding known anther development marker genes in cluster‐enriched genes or cluster‐specific genes, based on their expression patterns in *Arabidopsis* or other species. *HISTONE H2A 3* (*HTA3*) and *HTA5* accumulate during DSB (double strand breaks) formation,^[^ ^24^ ^]^ and were specifically expressed in cluster 12 (Figure 1E). *SHUGOSHIN 2* (*SGO2*), which maintains meiotic sister chromatid cohesion,^[^ ^25^ ^]^ was specifically expressed in cluster 13. Therefore clusters 12 and 13 were annotated as meiotic cells. The gene encoding tetratricopeptide repeat protein MALE‐STERILE 5 (MS5), required for cell cycle exit after meiosis II and for the transition to mitosis,^[^ ^26^, ^27^ ^]^ was specifically expressed in cluster 14, and indicative of microspores after meiosis. Based on the enrichment of *ABORTED MICROSPORES (AMS*),^[^ ^28^ ^]^ *MYB DOMAIN PROTEIN 35* (*MYB35*/*TDF1*),^[^ ^29^ ^]^ *ACYL‐COA SYNTHETASE 5* (*ACOS5*),^[^ ^30^ ^]^ the clusters 2, 5, 7, 10 were defined as tapetum cell. *GASSHO1* (*GSO1*) is specifically expressed in the middle layer in *Arabidopsis* ^[^ ^31^ ^]^ and was specifically expressed in cluster 4, defining it as middle layer cell. The development of endothecium cells is essential for anther dehiscence, with auxin and JA signaling in the endothecium involved in the control of anther dehiscence.^[^ ^32^, ^33^ ^]^ JA‐related and auxin‐related genes, such as *ALLENE OXIDE CYCLASE 4* (*AOC4*) and auxin‐responsive gene *GRETCHEN HAGEN 3* (*GH3*), were enriched in clusters 3, 6, and 11, and so these clusters were designated as endothecium cell. Because of the enrichment of *HOMEODOMAIN GLABROUS 2* (*HDG2*), *LIPID TRANSFER PROTEIN 6* (*LTP6*), and *ECERIFERUM 3* (*WAX2*),^[^ ^34^, ^35^, ^36^ ^]^ we annotated clusters 1 and 8 as epidermis cell. Phloem gene *PHLOEM PROTEIN 2‐A1* (*ATPP2‐A1*),^[^ ^37^ ^]^ NAC domain transcription factor *NAC DOMAIN CONTAINING PROTEIN 83* (*NAC083*) that regulates xylem vessel formation,^[^ ^38^ ^]^ *LACCASE 14* (*LAC14*) and *TERPENE SYNTHASE 21* (*TPS21*), reported to be expressed in the xylem and anther filaments,^[^ ^39^, ^40^ ^]^ were specifically detected in clusters 17, 15, 16, 9, respectively. These clusters were collectively referred to as vascular cells. The connective tissue of anthers lacks in‐depth studies and specific markers. Cluster 0 was enriched with some genes related to material transport, such as cation transporter *CATION EXCHANGER 3* (*CAX3*).^[^ ^41^ ^]^ Therefore, we tentatively presumed Cluster 0 to be connective cells. The diagrams in Figure 1E display the expression patterns of selected marker genes in different proposed anther cell types.

To further confirm the accuracy of cluster annotation, we performed RNA in situ hybridization assays on marker genes for each cell type (Figure 1F). The results showed that our presumed cell classification above was correct. For example, *3‐KETOACYL‐COA SYNTHASE 11* (*KCS11*), *PEROXIDASE 71* (*PRX71*), *AMS*, *HTA3*, *MS5* exhibited specific signals in the epidermis cell, endothecium cell, tapetum cell, meiotic cell, microspores after meiosis, respectively. *TPS21*, *NAC083* showed specific signals in vascular cells. *CAX3* showed strong signals in connective cells. Interestingly, *PEROXIDASE40* (*PRX40*), which was specifically expressed in the tapetum cells in *Arabidopsis*,^[^ ^42^ ^]^ was specifically expressed in the middle layer cell in cotton (Figure 1F). Therefore, based on the single‐cell transcriptional atlas we constructed, we uncovered a large number of cellular marker genes of each type of anther, filling gaps in knowledge for anther tissue marker genes. Due to the conserved nature of plant anther tissues, our atlas also should provide very useful information for subsequent studies on anthers of other species.

### 2.3 The Degradation of the Middle Layer Ends at Stage 11 Anther in Cotton

Meiosis is a vital life event for sexually reproducing organisms by generating new allele combinations through recombination,^[^ ^43^ ^]^ and the meiotic process and cell development are well understood.^[^ ^44^ ^]^ Since snRNA‐seq can explore the continuous differentiation trajectory of a developmental process,^[^ ^18^, ^45^ ^]^ we first used scRNA‐seq to deduce the developmental trajectory of anther meiotic cells to verify the feasibility of this method. Re‐clustering of clusters 12 and 13 representing the meiotic cells (Figure 1C) revealed seven sub‐clusters, named M1 to M7 (Figure S3A, Supporting Information). We ordered the seven sub‐cluster cells along a reconstructed trajectory of meiotic cell development by using monocle3 (Figure S3B, Supporting Information). Since *HTA3* was expressed in cluster 12 and *SGO2* was specifically expressed in cluster 13 (Figure 1E), and a large amount of DNA replication‐related genes were enriched in the M1 subcluster, we defined M1 as the root of the trajectory (Figure S3B, Supporting Information). Along with developmental trajectory, differential gene expression and module analysis were performed, uncovering substantial marker genes during meiosis, among of which are novel (Figure S3C and Table S3, Supporting Information). Functional gene set enrichment analysis was performed on genes within each module (Figure S3C, Supporting Information). Module 1 was mainly composed of M1 and M2 subpopulation cells and was enriched for DNA replication‐related pathways; Module 2, which contained a large number of M3 subpopulation cells, was enriched for chromosome condensation and nucleosome assembly pathways; Module 3, which was mainly composed of M4 subpopulation cells, was enriched for chromosome separation‐related pathways; Module 4 and Module 5, which contained a large number of M5 and M6 subpopulation cells, respectively, were enriched for sister chromatid separation‐related pathways during meiosis II; and Module 6, which was mainly composed of M7 subpopulation cells, was enriched for signal release and other related pathways. Based on the results of gene enrichment for each module, we classified the different modules into different processes of meiosis (Figure S3C, Supporting Information), mostly consistent with the classical meiotic process.^[^ ^44^ ^]^ Thus, the recapitulation of the meiotic process at the single‐cell level and the identification of specific genes along the developmental trajectory of meiotic cells not only provide clues for an in‐depth study of the meiotic process, but also further demonstrate the potency of the single‐cell transcriptome in probing cell developmental processes.

**Figure 2:**
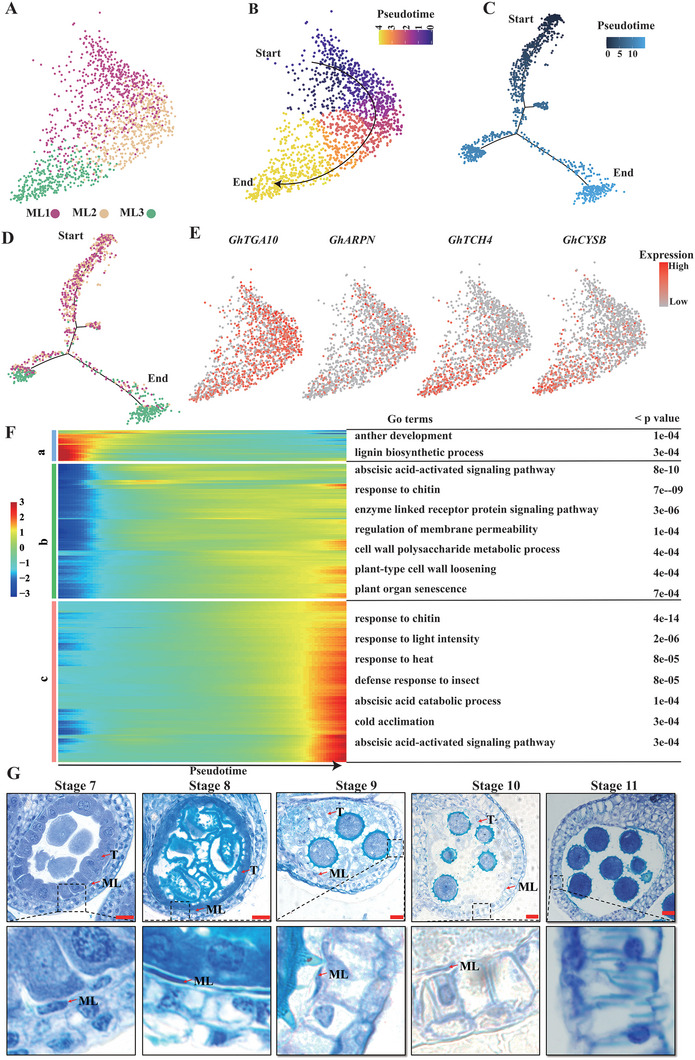
Developmental trajectory of middle layer.A) UMAP projections showing middle layer cells populations. ML1 to ML3, sub‐cell clusters. B) UMAP of the pseudotime trajectory of middle layer cells by using Monocle3. C,D) Simulation of the successive developmental trajectory of middle layer over pseudo‐time by using Monocle2. E) Expression patterns of *TGA10*, *ARPN*, *TCH4*, and *CYSB* over pseudo‐time. F) Heatmap showing hierarchical clustering of the expression of DEGs in each pseudotime cluster. GO terms and p value of each gene cluster are shown in the table on the right. G) Observation of the middle layer in cotton anther at stage 7 to stage 11. Scale bar, 20 µm. *TGA10*, *TGACG MOTIF‐BINDING PROTEIN 10*; *ARPN*, *PLANTACYANIN*; *TCH4*, *TOUCH 4*; *CYSB*, *CYSTATIN B*; ML, middle layer; T, tapetum.

The middle layer is the least characterized cell type in the anther lobe. Only a few genes are known to exclusively affect the development of the middle layer^[^ ^5^ ^]^ and a small number of genes have been reported to be specifically expressed in the middle layer.^[^ ^31^ ^]^ The middle layer is usually considered to be a short‐lived cell type that undergoes programmed cell death (PCD before the completion of meiosis (before stage 7).^[^ ^2^, ^5^ ^]^ To further understand the PCD process of the middle layer before meiosis, the developmental trajectory of the middle layer was analyzed. First, the middle layer cells were re‐clustered and divided into three sub‐cell clusters, named ML1 to ML3 (**Figure** **2**A). Second, we ordered the three sub‐cluster cells along a reconstructed trajectory of middle layer development by using monocle3 and monocle2 (Figure 2B–D). Inferred trajectories demonstrated gradual transitions from cells in ML1, ML2 to subcluster ML3 (Figure 2B–D). We tentatively defined ML1 as the root of this trajectory due to the lack of reported temporal marker genes in the middle layer. Along the developmental trajectory, we identified substantial novel genes specifically expressed within each pseudo‐temporal cluster (Table S4, Supporting Information), such as the *TGACG MOTIF‐BINDING PROTEIN 10* (*TGA10*) gene and the *PLANTACYANIN* (*ARPN*) gene, which regulate anther development in *Arabidopsis*.^[^ ^46^, ^47^ ^]^ These were highly expressed in ML1 and ML2 subpopulations, respectively, and the stress response factors *XYLOGLUCAN ENDOTRANSGLUCOSYLASE/HYDROLASE 22* (*TCH4*) and *CYSTATIN B* (*CYSB*)^[^ ^48^, ^49^ ^]^ were distinctly expressed in the ML3 subpopulation (Figure 2E; Table S4, Supporting Information). Signature genes of the pseudotime cluster a were enriched for “anther development”, however, signature genes of the pseudotime cluster b or cluster c were enriched for “abscisic acid‐activated signaling pathway”, “plant‐type cell wall loosening”, and some entries related to stress response (Figure 2F). The marker genes of pseudo‐temporal cluster a were enriched in gene ontology (GO) pathways such as “anther development”, indicating that the middle layer cells of pseudo‐temporal cluster a were still in the process of development, further proving that our previous definition of ML1 as the root of pseudotime trajectory was correct. The marker genes of pseudotime clusters b and c were enriched in “abscisic acid‐activated signaling pathway”, “plant‐type cell wall loosening”, and some GO pathways related to stress response (Figure 2F). From these results, we speculate that the middle layer cells in cotton anthers are not degraded after meiosis, but have just started the PCD process.

To test our conjecture, we observed the middle layer in cotton anther (Figure 2G). At stage 7, the morphology of the middle layer did not change significantly. As the anther developed, the middle layer started to become elongated and eventually disappear completely by stage 11 (Figure 2G), which was consistent with previous report.^[^ ^50^ ^]^ Interestingly, this phenomenon very similar to the PCD process of the tapetum, so there may be a synergistic mechanism for the disappearance of the middle layer and the tapetum in cotton.

### 2.4 Reconstruction of the Continuous Developmental Trajectory of Tapetum from a Single‐Cell Snapshot

**Figure 3:**
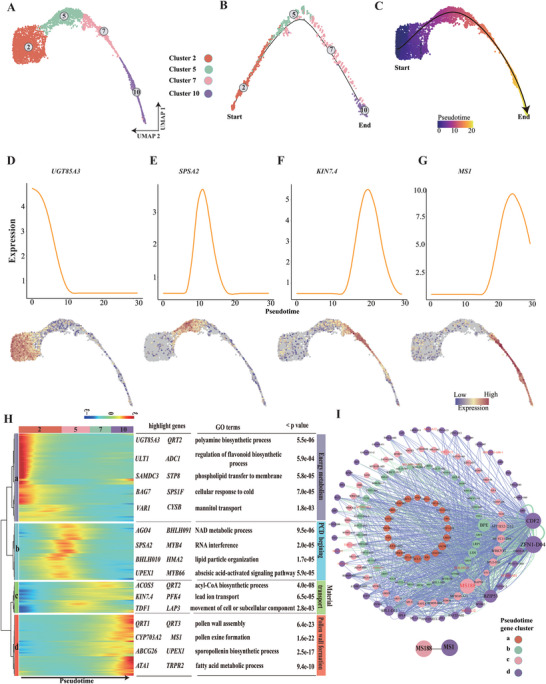
Developmental trajectory of tapetum cells. A) Uniform manifold approximation and projection (UMAP) showing tapetum cell populations (clusters 2, 5, 7 and 10). Each dot denotes a single‐cell. B) Simulation of the successive developmental trajectory of tapetum over pseudo‐time by using Monocle2. “Start” denotes the beginning of pseudo‐time. “End” denotes the ending of pseudo‐time. C) UMAP of the pseudotime trajectory of tapetum cells by using Monocle3. D–G) Expression patterns of *GLUCOSYL TRANSFERASE 85A3 (UGT85A3)*, *SUCROSE‐PHOSPHATE SYNTHASE A2 (SPSA2)*, *KINESIN 7.4 (KIN7.4)*, and *MALE STERILITY 1 (MS1)* over pseudo‐time. Color bar indicates the relative expression level. H) Heatmap showing hierarchical clustering of the expression of differentially expressed genes (DEGs) in each tapetum cell cluster along pseudotime. Representative cluster‐dependent genes, GO terms and p value of each gene cluster are shown in the table on the right. Color bar indicates the relative expression level. I) A gene regulatory network (GRN) built of 155 TFs expressed dynamically across tapetum pseudotime with a parameter cutoff of 0.1. Node size is equivalent to the number of predicted connections. Edge color represents activation (blue) or repression (green). Edge width represents the strength of the predicted connection.

The tapetum is vital for proper microspore development, providing metabolites for sporopollenin precursor formation to protect pollen.^[^ ^51^, ^52^ ^]^ However, the precise developmental process of the tapetum at the tetrad stages of anther is poorly understood. Thus, a subset of cells of tapetum (clusters 2, 5, 7, and 10) were selected to deduce the developmental trajectory of the tapetum by applying monocle2 and monocle3 (**Figure** **3**A).^[^ ^53^, ^54^ ^]^ Consistent with distribution distance on the UMAP, inferred trajectories demonstrated gradual transitions from cells in cluster 2 to cluster 10 (Figure 3B,C). The expression of genes (DYSFUNCTIONAL TAPETUM 1 (*DYT1*), *TDF1*, *AMS*, MALE STERILITY 188 (*MS188*), MALE STERILITY 1 (*MS1*)) in the classic genetic pathway for tapetum development and function validated our developmental trajectory (Figure S4A, Supporting Information).^[^ ^8^ ^]^ Along the developmental trajectory, numerous specifically expressed genes within each pseudotime cluster were identified, many of which were completely novel (Figure 3D–G; Table S5, Supporting Information). For example, genes associated with energy metabolism such as *UDP‐GLUCOSYL TRANSFERASE 85A3* (*UGT85A3*), encoding a UDP‐glucosyl transferase^[^ ^55^ ^]^ and *SUCROSE‐PHOSPHATE SYNTHASE A2* (*SPSA2*)^[^ ^56^ ^]^ and genes associated with response to stress such as *ARABIDOPSIS THALIANA PHYTOCYSTATIN 6* (*CYSB*) and *BCL‐2‐ASSOCIATED ATHANOGENE 7* (*BAG7*)^[^ ^49^, ^57^ ^]^ were prominently expressed in pseudotime clusters a and b (Figure 3D,E; Figure S4B, Supporting Information), which were enriched for “pyrimidine nucleoside triphosphate biosynthetic process”, “NAD metabolic process”, “cellular response to cold”, “abscisic acid‐activated signaling pathway” based on functional enrichment analysis (Figure 3H). Thus, the tapetum cells in pseudotime clusters a and b assembled at the beginning of pseudo‐time mainly might provide energy for the meiotic process and start the PCD process, which is similar to a process of response to stress.^[^ ^58^ ^]^ *KINESIN 7.4* (*KIN7.4*), a part of kinesin complex,^[^ ^59^ ^]^ was specifically expressed in pseudotime cluster c (Figure 3F), which was enriched for “movement of cell or subcellular component”, “lead ion transport”. This means that as the tapetum develops, the tapetum activates the expression of transporter‐like genes in order to transport more metabolites needed for development to the microspores. For tapetum cell in cluster d, a large portion of marker genes (*ATP‐BINDING CASSETTE G26* (*ABCG26*), *QUARTET 3* (*QRT3*), *CYTOCHROME P450 703A2* (*CYP703A2*), *MS1*) are involved in pollen wall formation (Figure 3G,H), such as MS1 which regulates the expression of key sporophytic pollen coat protein genes in *Arabidopsis*.^[^ ^60^ ^]^ GO terms “pollen wall assembly”, “pollen exine formation”, “sporopollenin biosynthetic process” also were enriched in cluster d (Figure 3H), implying that the tapetum cells in cluster d assembled at the end of pseudo‐time were mainly responsible for pollen wall formation. Combining scRNA‐seq information and pseudotime inference, we reconstructed the developmental progression of the tapetal cells and found strong heterogeneity of expression among tapetal cells at the tetrad stage, demonstrating a very rapid functional transformation of tapetal cells during this period.

Transcription factors (TFs) and interactions between TFs have been reported to be essential for tapetum development and function.^[^ ^8^, ^60^ ^]^ To elucidate the genetic coordination of TFs along the tapetum developmental process, we inferred a gene regulatory network (GRN) using a pipeline integrating the transformation of linear ordinary differential equations (ODEs) and linear regression (SCODE).^[^ ^61^ ^]^ Incorporating TF expression dynamics across pseudotime (Figure S5, Supporting Information), the resultant network reveals key players in this process and their regulatory interactions. Many TFs that have been reported in *Arabidopsis* to be involved in tapetum development, such as *AMS*, *TDF1*, *MS1*, and *MS188*,^[^ ^62^ ^]^ were in this network (Figure 3I), with *MS188* also being a highly connected central regulator (Figure 3I). Some connections between TFs such as connection between MS188 and MS1 in the network (Figure 3I), are consistent with previous reports,^[^ ^60^ ^]^ further indicating the accuracy of our network. What is more, the majority of highly connected central regulators have not been implicated in tapetum development, for example, *MYB DOMAIN PROTEIN 52* (*MYB52*), a highly linked gene in the network (Figure 3I), which negatively regulates pectin demethylesterification in *Arabidopsis* seed coat mucilage.^[^ ^63^ ^]^ Pectin demethylesterification is critical for normal anther development and pollen wall formation,^[^ ^64^ ^]^ implying that *MYB52* may be a key factor in the regulation of tapetum development. In addition, three *MYB4* homologous genes appeared inside the network, together with our previous study showing that overexpression of *MYB4* in cotton caused shrunken tapetum and microspore cells,^[^ ^13^ ^]^ it suggests that MYB4 also play a very important role during tapetum development. The pseudotime analysis of snRNA‐seq data and TF network indicate development is more gradual than previous data suggest and provides valuable new information on tapetum biology.

### 2.5 Chromatin Accessibility Illuminates Single‐Cell Regulatory Dynamics of Cotton Anthers

**Figure 4:**
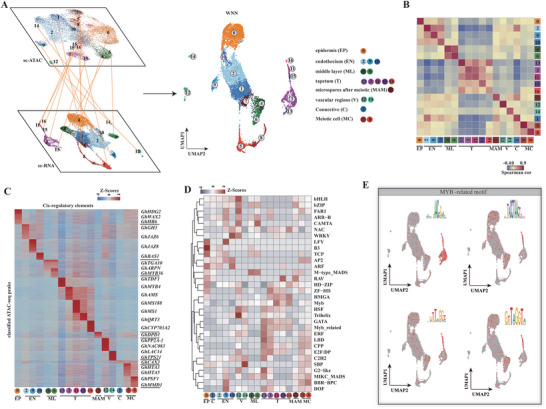
Single‑cell chromatin accessibility under NT in cotton anthers. A) UMAP plot of scRNA‐seq and scATAC‐seq data and their weighted mutual neighbor (WNN) data, as well as integrated WNN analysis. Cells are labeled by their scRNAseq‐annotated clusters. B) The cluster‐cluster correlation (Spearman correlation coefficient) between scRNA‐seq (bottom) and scATAC‐seq (right). The scATAC‐seq was calculated based on gene activity scores per cluster. The scRNA‐seq correlation was calculated based on gene expression per cluster. C) Heatmap of Z‐scores of cluster‐specific peaks derived from scATAC‐seq. D) The mean TF family motif enrichment (average deviation scores per cluster per TF family) across all clusters scale by row with z‐score. E) UMAP projection of scATAC‐seq profiles colored by chromVAR TF motif bias‐corrected deviations for the MYB‐related TFs. EP, epidermis; EN, endothecium; ML, middle layer; T, tapetum; MAM, microspore after meiotic; V, vascular region; C, connective; MC, meiotic cell.

In eukaryotes, gene expression is highly correlated with open chromatin.^[^ ^65^ ^]^ Chromatin profiles established at the single‐cell level by the single‐cell assay for transposase accessible chromatin using the sequencing technique scATAC‐seq can effectively reveal putative TF binding sites.^[^ ^66^ ^]^ To comprehensively elucidate the expression heterogeneity among anther cell types, we applied multi‐omics (ATAC+RNA) single‐cell sequencing to capture the nuclei from anthers at the tetrad stage as described above, using the 10X Multiome platform (Table S1, Supporting Information). 6107 cells were filtered under NT, and we obtained both expression and chromatin accessibility information (Table S1, Supporting Information). The cell types in NT samples were similar to these described above with regard to captured cell types (Figure S6A,B, Supporting Information), and the gene expression correlation was high (*R^2^* = 0.95 for the scaled expression) (Figure S6C, Supporting Information). Then, we used the mutual nearest neighbor algorithm (MNN) and weighted mutual neighbor algorithm (WNN) to integrate the horizontal and vertical multi‐omics data (**Figure** **4**A), and found that gene expression in scRNA‐seq was highly congruent with gene activity scores in scATAC‐seq. This further illustrates a strong correlation between chromatin accessibility and gene transcriptional activity (Figure 4B).

Furthermore, we identified numerous cell type‐specific accessible chromatin regions (ACRs) based on a total of 175253 ACRs across all clusters (Figure 4C; Table S6, Supporting Information). We annotated these cell type‐specific ACRs with the promoters of neighboring genes. To this end, we identified a list of marker genes with cluster‐specific ACRs. For example, the promoter of the epidermis marker gene *GhHDG2* contains an epidermis‐specific ACR, the promoters of *GhAMS* and *GhMS188* contain tapetum specific ACRs, and the promoters of *GhHTA3* contain meiotic cell specific ACRs. These results suggest that scATAC‐seq is also powerful for identifying different cell types and that differentiation of each cell type in anthers is associated with chromatin‐specific opening in each cell type.

Based on the identified ACRs in cotton anthers, the chromatin accessibility of *cis*‐elements that TF binding motifs under NT were measured using chromVAR, to elucidate the gene expression regulatory network responsible for cotton anther cell type differentiation as determined by the interaction between open chromatin and TF activity (Figure 4D). We revealed many cluster‐enriched TF motifs by calculating average deviation scores of each TF family across all clusters (Figure 4D). For example, TCP, B3 and LFY family TF motifs were enriched in epidermis; ARF, DOF family motifs were enriched in endothecium; SBP, bHLH, FAR1 family motifs were enriched in vascular regions; and M‐type MADS family motifs were enriched in middle layer cells (Figure 4D). The enrichment of TF motifs was coincident with the known regulators of cell identity, including the TCP family in epidermis development in leaves,^[^ ^67^ ^]^ the ARF family TFs in anther endothecium development,^[^ ^68^ ^]^ the SBP family in vascular development,^[^ ^69^ ^]^ and the MADS family in anther middle layer regulation.^[^ ^70^ ^]^ MYB‐related family motifs were enriched in all sub‐clusters belonging to tapetum, implying that MYB TFs play an essential role in regulating the development of the tapetum (Figure 4D,E). The above results show that each cell type has its own unique chromatin accessibility at the single‐cell level, providing an approach to elucidate the regulatory network of gene expression in the differentiation of cotton anther cells.

### 2.6 The Tapetal Cells Responsible for Pollen Wall Synthesis are Sacrificed Under HT

**Figure 5:**
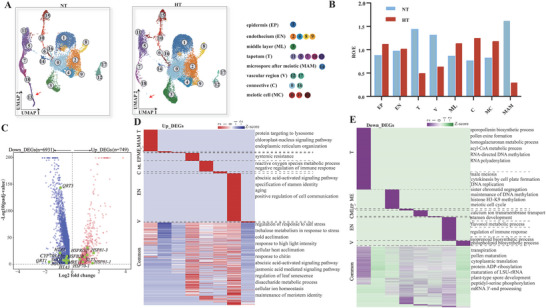
Construction of a single‐cell transcriptome atlas under HT of cotton anthers. A) UMAP plots of NT and HT cells after integration using Seurat. After integration, cells were clustered and labeled based on a previously annotated NT reference dataset. B) Preference of each cell type under HT stress. C) The number of up‐regulated and down‐regulated genes under HT was shown in a volcano plot. D) The above represents each cell type‐specific upregulated gene and the corresponding enrichment pathways. The below represents upregulated genes and the corresponding enrichment pathways in more than two cell types. Color bars indicate the z‐score of −log10 (*p*‐value). E) The above represents each cell type‐specific down‐regulated gene and the corresponding enrichment pathways. The below represents down‐regulated genes and the corresponding enrichment pathways in more than two cell types. Color bars indicate the z‐score of −log10 (*p*‐value). EP, epidermis; EN, endothecium; ML, middle layer; T, tapetum; MAM, microspore after meiotic; V, vascular region; C, connective; MC, meiotic cell; NT, normal temperature; HT, high temperature.

HT is an abiotic stress that can cause severe crop yield reduction,^[^ ^71^ ^]^ but whether different anther cell types respond to HT similarly has not been studied. We collected anthers at the tetrad stage in the field after seven days of heat stress and isolated nuclei for single nucleus RNA‐Seq (Figure S7A, Supporting Information). For the HT treated sample, we captured 15906 high quality cells (Table S1, Supporting Information). We integrated NT and HT samples using Seuart and yielded corresponding clusters in NT and HT cells based on conserved maker genes (Figure S8, Supporting Information). Strikingly, we found that the number of tapetum cells decreased noticeably under HT. In particular, the tapetum cells responsible for pollen wall synthesis disappeared completely under HT, as observed by UMAP plots (**Figure** 5A). Further, the ratio of the observed and expected cell numbers (R_O/E_) for each cell type under NT and HT was calculated using a chi‐square test (Figure 5B), and it was found that the cell types displayed significant distinct preferences between the NT and HT samples. For example, the number of tapetum cells and microspores were significantly reduced, while the number of epidermal cells and connective tissue cells were increased in the single‐cell transcriptome atlas under HT (Figure 5B), implying different cell types respond to HT differently. Therefore, differential expression analysis of different cell types between NT and HT was performed. Overall, the number of down‐regulated genes (n = 6931) was substantially higher than the number of up‐regulated genes (n = 749) under HT (Figure 5C), consistent with previous reports in *Arabidopsis*.^[^ ^72^, ^73^ ^]^ There were stress‐responsive genes among the up‐regulated genes, such as *HEAT SHOCK PROTEIN*, which was strongly expressed across all clusters (Figure 5C; Figure S7B, Supporting Information). Moreover, half of the upregulated genes (n = 353) were upregulated in more than two cell types, and the gene enrichment pathways for each cell type‐specific upregulated gene cluster essentially resembled stress response pathways, such as “cellular heat acclimation”, “aging”, and others (Figure 5D). However, among the downregulated genes under HT, cell‐type specific genes were enriched, such as tapetum‐specific gene *QRT3*, meiotic cell‐specific gene *HTA5*, based on functional enrichment analysis (Figure 5C–E). For instance, “sporopollenin biosynthetic process”, “pollen exine formation”, and “homogalacturonan metabolic process” were overwhelmingly repressed under HT in tapetal cells; “DNA replication”, “sister chromatid segregation”, “male meiosis” pathways were down‐regulated under HT in meiotic cells; and “calcium ion transmembrane transport” was inhibited in middle layer cells (Figure 5E). These results suggest that in response to HT stress, anthers globally upregulate stress response genes and specifically downregulate genes involved in growth and development in different cell types, presumably to enhance resistance to HT.

### 2.7 Closure of Chromatin Accessibility of Pollen Wall Synthesis Genes Under HT Caused Abnormal Pollen Wall Development

**Figure 6:**
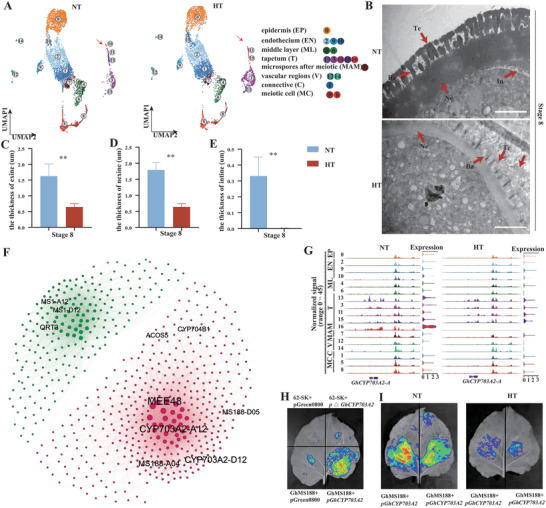
The tapetal cells responsible for pollen wall synthesis are the most sensitive to HT. A) UMAP visualization of the integration of scRNA‐seq scATAC‐seq under NT and HT base on the WNN graph. B) Transmission electron microscopy analysis of pollen walls at stage 8 under NT and HT. Scale bar, 2 µm. C) The exine, D) nexine, and E) intine wall thickness of pollen walls under NT and HT at stage 8. The *P*‐value was calculated by using the Student *t*‐test (n >24). The error bars represent standard deviations (SDs). ^**^ *p* <0.01. F) Gene co‐expression network for tapetal cells that disappeared under HT. The marked genes represent genes that have been previously reported to be associated with pollen wall formation. Different colors represent different modules. G) Visualization of chromatin accessibility tracks of the *Gh* *CYP703A2* locus across all clusters. H) Luminescence imaging of transient dual‐luciferase reporter assay between GhMS188 and *Gh* *CYP703A2* promoter or *Gh* *CYP703A2* promoter without MYB‐binding sites. Luminescence signals on *N. benthamiana* leaves were visualized using a cryogenically cooled CCD camera. p ***Δ*** CYP703A2 represents the *Gh* *CYP703A2* promoter without MYB‐related binding elements. I) Associations of GhMS188 with *Gh* *CYP703A2* promoter in LUC assays in *N. benthamiana* leaves under NT and HT. EP, epidermis; EN, endothecium; ML, middle layer; T, tapetum; MAM, microspore after meiotic; V, vascular region; C, connective; MC, meiotic cell; In, intine; NE, nexine; Ba, bacula; Te, tectum; NT, normal temperature; HT, high temperature.

To explore the chromatin accessibility dynamics under HT at the single‐cell level, we conducted a study combining multi‐omics data under HT. We first analyzed UMAP plots under NT and HT based on the WNN graph (**Figure** **6**A). Consistent with the expression changes under HT in scRNA‐seq (Figure 4A), tapetal cells responsible for pollen wall synthesis also disappeared from the WNN graph under HT (Figure 6A), implying not only that both chromatin accessibility and expression of genes in the tapetal cells responsible for pollen wall synthesis were inhibited under HT, but also that the pollen wall synthesis process was extremely sensitive to HT. To further confirm this possibility, we observed by transmission electron microscopy the pollen wall in the stage 8 anther (the intact pollen wall has been formed during the stage) under NT and HT (Figure 6B). Intriguingly, the exine under HT was significantly reduced in thickness and lacked an intact bacula and tectum (Figure 6B,C), and there was an accumulation of free material outside the pollen wall (Figure 6B), presumed to be a sporopollenin‐like substance. The nexine was also significantly reduced in thickness and the intine essentially disappeared under HT (Figure 6D,E). In general, HT stress specifically down‐regulated the growth and development pathways of each cell type, and the pollen wall synthesis pathway responsible for tapetum cells was most severely inhibited in the tetrad stage anther.

To further investigate the molecular mechanism of the degradation of tapetal cells associated with pollen wall synthesis under HT, we performed a gene coexpression network analysis of the tapetal cells responsible for pollen wall formation under NT, based on weighted gene coexpression analysis (Figure 6F; Table S7, Supporting Information). We uncovered many potential hub genes that regulate pollen wall formation in modules, included many already reported to be related to pollen wall synthesis genes, such as *MATERNAL EFFECT EMBRYO ARREST 48* (*MEE48*), *Gh*CYP703A2, *MS188*, *QRT3*, *ACOS5* ^[^ ^7^, ^74^ ^]^ (Figure 6F; Table S7, Supporting Information). Therefore, we believe that the reason for abnormal development of pollen wall under HT is that HT inhibits the chromatin accessibility activity of these hub genes, leading to their expression being silenced under HT. To verify this speculation, we selected several hub genes for visualization of chromatin accessibility (Figure 6G; Figure S9, Supporting Information). The result shows that the chromatin accessibility near genes associated with pollen wall formation in modules is substantially decreased inside other clusters of tapetum, in addition to the absence of signal in cluster 16 under HT (Figure 6G; Figure S9, Supporting Information). This evidence demonstrates that HT indeed causes a reduced chromatin accessibility of genes related to pollen wall synthesis, repressing their expression.

In order to further explore the regulatory mechanism of the down‐regulation of gene expression caused by the decrease of chromatin accessibility activity of pollen wall synthesis‐related genes under HT. We analyzed the chromatin accessibility of 465 TF motifs in all clusters under HT conditions (Figure S10, Supporting Information). Interestingly, the chromatin openness of motifs of different TF families showed differences in different anther cells under HT (Figure S10, Supporting Information). For example, the chromatin openness of motifs of MYB‐class transcription factors, which are highly enriched in the tapetal cells (Figure 5D), was severely reduced in tapetal cells (including tapetal cells associated with pollen wall synthesis) under HT (Figure S10, Supporting Information). Additionally, we also discovered that the promoters of hub genes associated with pollen wall synthesis have the highest abundance of MYB‐related motifs (Figure S10C, Supporting Information). These results not only suggest that the heterogeneity of different cells in response to HT may be the result of differential TF regulation, but also that the closure of chromatin accessibility of TF motifs such as MYB under HT will lead to the inability of TFs such as MYBs to regulate the expression of pollen wall synthesis‐related genes normally under HT. In order to further validate this hypothesis, we used the R2R3 MYB gene family member *GhMS188* and *Gh* *CYP703A2* in the co‐expression network for verification. The results showed that GhMS188 can activate *Gh* *CYP703A2* expression by binding to the MYB binding site on the *Gh* *CYP703A2* promoter (Figure 6H). However, the ability of GhMS188 to activate *Gh* *CYP703A2* substantially weakened under HT (Figure 6I). This result indirectly inferred that the chromatin accessibility of MYB binding sites on *Gh* *CYP703A2* may decrease under high temperature, leading to the weakened activation capacity of GhMS188 for *Gh* *CYP703A2*. Therefore, the decreased chromatin accessibility of pollen wall synthesis genes under HT might cause MYB and other TF unable to regulate the expression of pollen wall synthesis genes properly, ultimately resulting in abnormal pollen wall development.

### 2.8 *QRT3* and *CYP703A2* are Key Core Genes Lost in Degrading Tapetum Cells Under HT Stress

**Figure 7:**
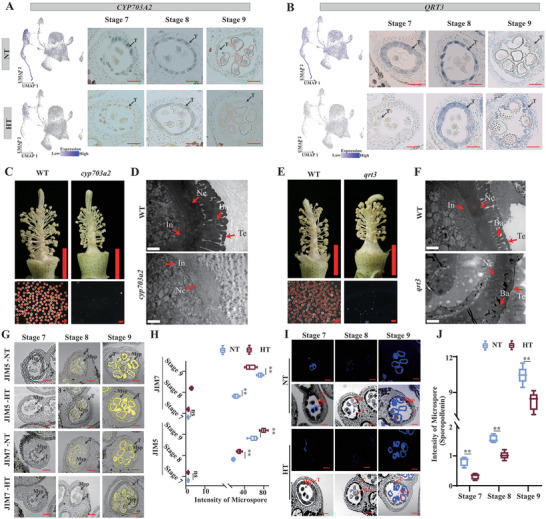
HT inhibits the expression of *QRT3* and *CYP703A2* and causes male sterility. A,B) UMAP plots above showed the expression of *CYP703A2* (A) and *QRT3* (B) under NT and HT. The right panel represents the RNA in situ hybridization of *CYP703A2* (A) and *QRT3*(B) under NT and HT at stage 7 to 9. Scale bar, 50 µm. C,E) Male Phenotypes of *cyp703a2* (C), *qrt3* (E), and WT plants under NT. Red pollen grains represent fertile and white pollen grains represent sterile. Scale bars above = 5 mm. Scale bars below = 200 µm. D,F) Transmission electron microscopy analysis of pollen walls from the *cyp703a2* (D), *qrt3* (F), and WT plants under NT at stage 8. Scale bar, 2 µm. G) Immunofluorescence studies of pectin of anther under NT and HT at stage7 to 9. Sections were stained with antibodies against de‐esterified pectin (JIM5) and esterified pectin (JIM7). Scale bar, 50 µm. H) Immunofluorescence signals intensity of microspore in (H) measured by ImageJ under NT and HT. The *P*‐value was calculated by using the Student *t*‐test (n >10). ns, not significant. ^**^ *p* <0.01. I) Sporopollenin autofluorescence in NT and HT anther sections at stage 8, stage 9, and stage 10. Scale bar, 50 µm. J) Fluorescence signals intensity of microspore in (G) measured by ImageJ under NT and HT. The *P*‐value was calculated by using the Student *t*‐test (n >10). ^**^ *p* <0.01. In, intine; NE, nexine; Ba, bacula; Te, tectum; T, tapetum; Msp, microspore; NT, normal temperature; HT, high temperature.

*QRT3* and *CYP703A2* appeared in the network associated with pollen wall synthesis (Figure 6F), and are essential for normal cotton pollen wall development.^[^ ^64^, ^75^ ^]^ We extracted the expression of these two genes from our data and found both genes were mainly expressed in those tapetal cells lost under HT (**Figure** **7**A,B). RNA in situ hybridization experiments showed that the expression of *CYP703A2* and *QRT3* was high at stages 7 and 8 under NT, but both were very obviously and strongly repressed at stage 7 in HT‐sensitive line H05 under HT (Figure 7A,B). Knockout mutants of *CYP703A2* and *QRT3* both showed a very pronounced male sterile phenotype with indehiscent anthers and lack mature pollen (Figure 7C,E). Transmission electron microscopy revealed that compared with wild type, *cyp703a2* failed to form a normal sexine with tectum and bacula (Figure 7D), whereas *qrt3* pollen possessed a very smooth sexine without spines (Figure S11, Supporting Information),^[^ ^65^ ^]^ with a significantly thickened nexine, and lacks intine (Figure 7F). These results indicate that both *CYP703A2* and *QRT3* have a positive regulatory effect on pollen wall synthesis.

Therefore, HT stress can inhibit normal pollen wall formation by suppressing the expression of *CYP703A2* and *QRT3*. QRT3 is a polygalacturonase involved in the pectin metabolic pathway,^[^ ^64^ ^]^ while *CYP703A2* is important for the synthesis of sporopollenin.^[^ ^75^ ^]^ We performed immunofluorescence studies of pectin/homogalacturonan components and fluorescence detection of sporopollenin. Immunofluorescence studies showed that the pectin signal accumulated in tapetum and microspores at stages 8 and 9 both under NT and HT. JIM5 was used to detect de‐esterified homogalacturonan, which is the substrate of QRT3, and it significantly accumulated in tapetum under HT compared to NT at stage 9 (Figure 7G,H). This is consistent with the view that the excessive accumulation of JIM5 antigen leads to male sterility,^[^ ^64^ ^]^ indicating that HT affects the accumulation of de‐esterified pectin, which leads to male fertility. In contrast, the content of esterified homogalacturonan in the tapetum and microspores at stages 8 and 9 was reduced under HT (the fluorescence signal of JIM7 was significantly weakened) (Figure 7G,H). On the other hand, sporopollenin fluorescence on microspores was also significantly reduced in stage 7–9 anthers in HT‐sensitive line H05 under HT (Figure 7I,J). These results demonstrate that HT inhibits the expression of *QRT3* and *CYP703A2*, affecting pectin metabolism and sporopollenin synthesis, respectively, and resulting in abnormal pollen wall development and male sterility.

## 3 Discussion

Revealing the mechanisms of plant development can not only help our understanding of the evolutionary process of life, but also can accelerate the development of agricultural science. scRNA‐seq has been used in crops as a powerful tool to interrogate the development of various cell types in plant tissues.^[^ ^18^, ^20^, ^76^ ^]^ The construction of a single‐cell transcriptional atlas of the rice root tip has laid a good foundation for resolving the molecular mechanisms of rice root development, engineering root systems, and improving nutrient uptake capacity.^[^ ^18^ ^]^ The construction of single‐cell expression profiles of cotton ovules provides a theoretical framework to explore fiber development and improve fiber quality and yield.^[^ ^20^ ^]^ As the male organ of flowering plants, the anther is important in providing male gametes required for the fertilization process. For a more rationale molecular breeding strategy, it is therefore essential to understand the mechanisms involved. This involves defining the major cell types within the anther at the molecular level. Although some genes regulating anther development have been described in *Arabidopsis* and rice at the tissue level,^[^ ^7^ ^]^ a systematic understanding of spatio‐temporal anther development is still limited. The single‐cell resolution profile of cotton anther gene expression presented here provides a valuable resource for defining different anther cell types. Based on our scRNA‐seq and cell clustering, we successfully identified eight major cell types at the tetrad stage of cotton, which could be further classified into 18 subcell types (Figure 1C). Our results demonstrate the transcriptional heterogeneity amongst anther cells. Importantly, our study revealed specific marker genes for the eight anther cell types, filling a significant knowledge gap (Figure 1E; Table S2, Supporting Information). For example, specific marker genes have been lacking in the epidermis and endothecium at early stages of anther development, and middle layer, connective, and vascular tissue. Our in situ hybridization experiments validated the accuracy of our single‐cell expression profiles (Figure 1F). Pseudotime analyses revealed that the expression patterns of tapetal cells at the tetrad stage showed significant heterogeneity, and tapetal cells from different pseudotime clusters had different biological functions. By constructing the regulatory network of transcription factors during tapetal development, some important transcription factors such as *MYB4*, *MYB52*, and *ZFN1* were identified. Based on the pseudotime analysis and high‐precision semi‐thin section results of the middle layer, we propose that the degradation of middle layer cells in cotton is not complete by stage 7 after meiosis, but was complete by stage 11 of anther development, in coordination with the tapetal cells. Our study therefore provides a basis for subsequent anther development studies. Nevertheless, we found some differences in the expression patterns of homologous genes in different species. For example, *PRX9* and *PRX40* are specifically expressed in the tapetum in *Arabidopsis*,^[^ ^42^ ^]^ but are specifically expressed in the middle layer in cotton (Figure 1E,F). This phenomenon implies that there may be some divergence in anther development in different species, which needs to be noted in future functional genomic studies.

The single‐cell transcriptional atlas of the cotton anther reveals that the same type of cells can be classified into many clusters, indicating that the same type of cells belong to different developmental stages or that there are many subclasses of the same type of cells. Among them, clusters 2, 5, 7, and 10 belonging to the tapetal cells were separated from other clusters on the UMAP plot, indicating the unique transcriptional pattern of tapetal cells (Figure 1C). The pseudotime trajectory analysis of tapetal cells revealed that the cells in these four clusters are at different developmental stages (Figure 3). We also found that endothecium cells were divided into clusters 3, 6, and 11 (Figure 1C), indicating heterogeneity. Some studies have identified a new type of endothecium cells, named interendothecium cells because they are located on the inner side of the anther lobe and have a large vesicle and lack chloroplasts.^[^ ^77^ ^]^ Some studies have revealed that interendothecium cells may have an expression pattern that is inconsistent with endothecium cells.^[^ ^78^ ^]^ Therefore, clusters 3, 6, and 11 belonging to endothecium may contain interendothecium cells, and we may subsequently try to identify interendothecium and uncover their specific transcriptional markers. On the other hand, all cell layers of the anther develop from the differentiation of the stamen primordia.^[^ ^2^ ^]^ Although we were unable to harvest enough stamen primordia for single‐cell transcriptome sequencing, a more precise description of the trajectory of stamen primordia differentiation into specific anther wall cells in the future will help us to better understand early stages of anther differentiation.

Gene expression is regulated by a variety of factors including chromatin accessibility, transcription factor expression and availability and epigenetic modifications. The chromatin accessibility of TF motifs can influence the ability of TFs to regulate target genes. To gain more insight into the mechanisms of gene expression regulation between different anther cells, we applied single‐cell multi‐omics sequencing to obtain both expression and chromatin accessibility information (Figure 4A). A unique landscape of chromatin accessibility was established and different TF regulation mechanisms were elucidated for each anther cell type (Figure 4C,D). After combining expression and chromatin accessibility, we found a huge effect of chromatin accessibility on the transcriptional activity of cotton anther genes (Figure 4B). Although we established a joint map for anther single‐cell transcriptome and chromatin accessibility, plant development has complex mechanisms that need to be elucidated by including other dimensions. Therefore, combining more omics technologies to resolve plant growth and development processes will ultimately improve the accuracy and efficiency of breeding. However, combined multi‐omics analysis at the single‐cell level is rarely applied in plants, though it has been widely used in animals. For example, high‐throughput single‐cell transcriptome and spatial transcriptome has been used to construct a large‐scale single‐cell spatio‐temporal atlas of human intestinal development and to define the cells and locations associated with rare developmental intestinal diseases.^[^ ^79^ ^]^ Single‐cell multi‐omics studies also reveal the mystery of intermediate neuronal differentiation processes in the cerebral cortex.^[^ ^80^ ^]^ Thus, the future use of single‐cell multi‐omics can be expected to lead to important new discoveries in the field of plant science.

**Figure 8:**
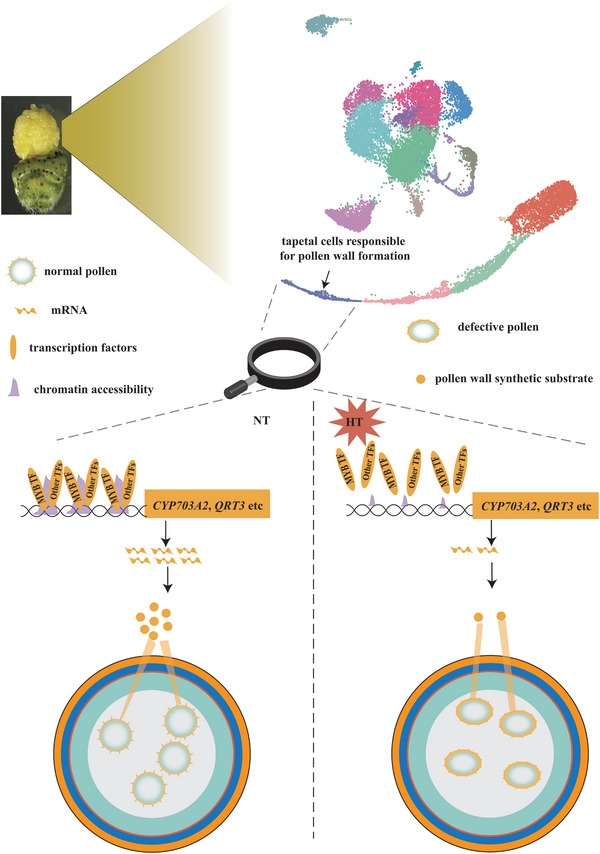
Model of the effect of HT on pollen wall formation. This model shows chromatin accessibility of genes specifically expressed in the tapetum cells responsible for pollen wall synthesis was closed under HT, resulting in the silencing of these genes due to the inability of transcription factors like MYB to bind to their promoter regions to regulate their expression, ultimately leading to abnormal pollen wall formation under heat stress. NT and HT: normal temperature and high temperature, respectively.

We constructed a joint anther map of single‐cell expression and chromatin accessibility not only under normal conditions, but also under HT stress (Figure 6A), and systematically described the gene expression and chromatin accessibility. Different cell types respond differently to HT (Figure 5B–E), with the tapetum cells responsible for pollen wall synthesis being the most sensitive to HT (Figure 5A). The chromatin accessibility of genes specifically expressed in the tapetum cells and responsible for pollen wall synthesis is closed under HT, resulting in the silencing of these genes due to the inability of transcription factors like MYBs to bind to promoter regions to regulate their expression (Figure 6A,G–I; Figure S10, Supporting Information). This ultimately leads to abnormal pollen wall formation under heat stress and male sterility (Figure 6B). We also generated a model to depict these results (**Figure** **8**). Although stress‐responsive genes such as *HSP*s vary uniformly across all cell types (Figure 5D; Figure S7B, Supporting Information), development‐related genes have different expression patterns in different cell types under HT (Figure 5E). This information means that we can eventually generate heat‐tolerant crops by genetically manipulating the relevant cell types. For example, the expression of pollen wall synthesis‐related genes such as *QRT3*, *CYP703A2* is severely suppressed under HT in HT‐sensitive line, resulting in abnormal pollen wall development and eventual male sterility. However, if we can optimize these genes specifically expressed in the tapetum by adding promoters that are induced by HT so that they are not repressed under HT stress, it may be possible to generate heat‐tolerant crops.

## 4 Conclusion

In this study, we constructed the first single‐cell transcriptome atlas and chromatin accessibility survey in the anther at the tetrad stage under NT and HT. We described the specific expression and epigenetic profile of each cell type in anthers under NT, providing strong evidence for functional differentiation among anther cells. The dynamics of expression at the single‐cell level during the development of meiosis, tapetum, and middle layer cells were depicted at the tetrad stage. Heterogeneity among cell types in the response to HT was also revealed. Tapetal cells, responsible for pollen wall formation, are most sensitive to HT; *QRT3* and *CYP703A2*, specifically expressed in the tapetal cells, are core factors in response to HT. Our results not only provided a great deal of clues for the study of plant anther development, but also provided ideas for future improvement of heat‐tolerant plants.

## 5 Experimental Section

### Plant Materials and Growth Conditions

The cotton (*Gossypium hirsutum*) line H05 used in this study was grown in the field at Huazhong Agricultural University, Wuhan, China. The stage 7 anthers (tetrad stage) were harvested from 6–7 mm buds under 29–35 °C daytime and 25–27 °C nighttime temperatures, considered as normal temperature (NT) samples. HT stress begins to occur when cotton reaches full bloom (when there were buds at different stages of development). Anthers at stage 2–3 grown at 38–40 °C in the daytime and 28 to 31 °C at night for 7 days were developed into stage 7 (tetrad stage) and collected as HT‐stressed sample (Figure S12, Supporting Information). After collection, anthers at tetrad stage were immediately frozen in liquid nitrogen and stored at −70 °C until use.

### Preparation of Anther Samples for ScRNA‐Seq and 10X Multiomic

Briefly, cotton anthers were chopped with NI buffer (20 mm hepes (pH 8.0), 250 mm sucrose, 1 mm MgCl_2_, 5 mm KCl, 40% glycerol, 0.25% TrixtonX‐100, 0.2 U µL^−1^ Protector RNase Inhibitor) and then resuspended with NI buffer into 2 mL tubes placed on ice and incubated on a rocking shaker for 5 min. Next, samples were filtered through a pre‐wetted (using NI buffer) 40 µm strainer laid on top of a 2 mL tube placed on ice. Then samples were centrifuged at 5000 rpm for 5 min at 4 °C. After centrifugation, the supernatant was carefully removed, and the pellet resuspended in 400 µL 1X PBS buffer with 0.2 U µL^−1^ Protector RNase Inhibitor. The suspension was stained with DAPI to a final concentration of ≈1 µm and loaded into a FACSAria II flow cytometer. Nuclei were sorted for each sample across different catch tubes containing 200 µL 1X PBS. Isolated nuclei were spun down (5 min, 500 rcf) in a swinging‐bucket centrifuge, resuspended in 10 µL 1X PBS, pooled, and then visualized on a hemocytometer with a fluorescent microscope. Nuclei suspensions were then spun down (5 min, 500 rcf) and resuspended in diluted nuclei buffer (10X Genomics). scRNA‐seq libraries were constructed following the 10X Genomics Chromium Single‐Cell 3 v 3 protocol without modification. Multiomic libraries were prepared using the Single‐Cell Multiome ATAC + Gene Expression kit (10X Genomics).

### Single‐Cell RNA‐Seq Data Preprocessing

The cotton mitochondrial genome was merged with the nuclear TM‐1 genome (HAU‐AD1_v1.1).^[^ ^81^ ^]^ The reference genome was built with the “cellranger mkref” function in the Cell Ranger (version 6.1.2). Then raw data were mapped to the reference genome to create single‐cell gene counts by using “cellranger count” function in the Cell Ranger with “–id, –transcriptome, –fastqs, –sample and –r^2^‐length = 98” parameters. The gene‐cell matrices (named “filtered_gene_bc_matrices” by 10X Genomics) were served as processed raw data for further analyses.

DoubletFinder software (v.2.0.3) was used to detect doublet cells in each scRNA‐seq dataset.^[^ ^82^ ^]^ The doublet cell prediction requires three input parameters: the expected number of real doublets (nExp), the number of artificial doublets (pN), and the neighborhood size (pK). For nExp, the standard Seurat processing pipeline was used up to the clustering stage with low cell cluster number resolution (resolution = 0.5). The cluster labels of cells were used as “annotation” data to model the proportion of homotypic doublets. The doublet proportion was estimated by N/100 000 (N, the number of cells). The nExp value was adjusted according to the proportion of homotypic doublets and doublet proportion. pN was a ratio used to define the number of artificially generated doublets based on the total number of cells. pN was set to 0.25. To identify the optimal pK value, the pre‐processed Seurat data were loaded into the “paramSweep_v3 (PCs = 1:15)” function and subsequently fed into the “summarizeSweep” and “find.pK” functions. A single and easily discernible maximum of pK value was selected as the optimal pK parameter. Finally, the doublet cells were predicted with the pre‐processed Seurat data using the “doubletFinder_V3” function and the defined values of nExp, pN, and pK as described above. The proportion of artificial nearest neighbors (pANN) for each cell was computed. The doublet threshold of pANN was generated based on the expected number of doublet cells (nExp) to generate the final doublet predictions. Cells that were flagged as singlets were kept for further downstream analysis.

### Data Integration, Clustering, and Annotation of ScRNA‐Seq

Downstream analyses were mainly carried out using the Seurat package (v.4.1.1)^[^ ^83^ ^]^ as previously described.^[^ ^18^ ^]^ In brief, quality control procedures were performed by filtering out low‐quality cells and genes, and data were normalized using the “NormalizeData” function (LogNormalize method, scaling factor of 10 000). Then, variable genes were detected with the “FindVariableGenes” function (vst method, 2000 features), data were scaled with the “ScaleData” function, PCA analysis was performed with the “RunPCA” function (100 principal components), statistical significance of PCA scores was determined by the “JackStraw” function, the SNN graph was constructed, cells were clustered based on Louvain (“FindNeighbors” and “FindClusters”), and data were visualized with non‐linear dimensional reduction algorithms (“RunTSNE” and “RunUMAP”).

Low‐quality cells and genes were filtered based on the following four criteria: 1) only cells with the expressed gene number between 500 and 6000 were considered; 2) cells with Unique Molecular Identifiers (UMIs) above 40 000 or below 500 were excluded; 3) genes expressed in fewer than three cells were filtered out; 4) the percentage of mitochondrial UMIs was no more than 10%. Batch effects between samples were corrected using the “RunHarmony” function.^[^ ^84^ ^]^ The final integrated data were clustered and visualized based on harmony dimensionality reductions.

To mitigate the effects of cell cycle heterogeneity on cell clustering, the cell cycle score of each single‐cell was calculated using *Arabidopsis* cell cycling orthologous genes by using the “CellCycleScoring” function. These cell cycle effects were then regressed out using the “vars.to.regress” parameter in the “ScaleData” function.

Cluster‐enriched genes were computed with Seurat's “FindAllMarkers” function using the following parameters: a Wilcoxon Rank Sum test; above 1.5‐fold difference (logfc.threshold = 0.58) between the two groups of cells; test genes that had a minimum fraction of at least 0.1. Cell clusters were assigned by the resultant cluster‐enriched genes with known functions and expression patterns. To identify cluster‐specific marker genes, the following parameters were applied: the log2 fold change of genes was >0.25, and the proportion of marker genes expressed in cells among all other clusters (PCT2) was less than 10%.

### Reconstruction of the Cell Developmental Trajectory

The Monocle 2 (v.2.22.0) and Monocle 3 (v.1.0.0) R package was used to construct the cell developmental trajectory.^[^ ^53^, ^85^ ^]^ The subsets of raw data of meiotic cells, tapetal cells, middle layer cells were used to explore the pseudotime developmental trajectory. For Monocle2, the variable genes were identified based on the “dispersionTable” function (q <0.001). The “reduceDimension” (set max_ components = 2, method = DDRTree) function analyzed the dimensional reduction clustering. The pseudotime transition of cells was displayed by “orderCells”. “plot_pseudotime_heatmap” was used to cluster and visualize the DEGs along the trajectory. For Monocle3, the visualization of developmental trajectory was shown on UMAP plot. The root node was defined by the time of the biological event. The variable genes were identified based on the “Track_genes” function (q <0.001). The DEGs in each pseudotime modules were identified based on the “find_gene_modules” (resolution = 1 × 10^−2^, cores = 10). Gene ontology (GO) analysis was applied by using clusterProfiler R package.^[^ ^86^ ^]^

### Multiome Data Processing

The reference genome was made by “cellranger‐arc mkref” function in cellranger‐arc (10 X Genomics, v.2.0.1). Multiome raw data were aligned to the reference genome and quantified using “cellranger ‐arc count” (10X Genomics, v.2.0.1).

The RNA count data were further processed using “Seurat” as described above. scATAC‐seq analysis was carried out in the Signac package as previously described.^[^ ^83^, ^87^ ^]^ The study only retained cells with 1000–25 000 fragment counts in peak regions, a fragment proportion >15% in peak regions, and a calculated nucleosome signal of <10. To reduce the dimensionality of the scATAC‐seq fragment matrix, latent semantic indexing (LSI) was performed. The formula for calculating the term frequency‐inverse document frequency (TF‐IDF) of the peak matrix is: Each peak's accessibility in each cell was divided by the total accessibility in the cell (“term frequency”) and multiplied by the inverse accessibility of the peak in the cell population. This step “upweights” the contribution of highly variable peaks and downweights the contribution of peaks accessible in all cells. These values were then multiplied by 10 000 and logarithmically transformed in the TF‐IDF matrix, adding a pseudocount of 1 to avoid computing the logarithm of 0. Next, the TF‐IDF matrix was decomposed via SVD to return LSI components, and the scaling LSI loadings for each LSI component were normalized to have mean 0 and standard deviation 1.

Peak calling was then performed from all cells of each cluster using MACS2 (V2.2.7.1).^[^ ^88^ ^]^ To identify cluster‐specific accessible chromatin regions (ACRs), the following parameters were used: Log‐likehood ratio test; the log2 fold change of ACRs was >0.25 and the minimum proportion of ACRs expressed in cells among all clusters was >10%. ChromVAR (v.1.16.0)^[^ ^89^ ^]^ was used to obtain TF accessibility profiles using position weight matrices of *Arabidopsis* from the JASPAR 2020 database.^[^ ^90^ ^]^ Gene activity scores were computed using Signac package.^[^ ^87^ ^]^

### Integration of Seurat Objects Under Different Treatments

Data were integrated following the Seurat integration pipeline.^[^ ^87^ ^]^ Anchors between the NT scRNA‐seq and HT scRNA‐seq datasets were identified using canonical correlation analysis (CCA), NT samples as the reference. These anchors were used to transfer cell‐type labels from NT scRNA‐seq to HT scRNA‐seq. Similarly, CCA was used to integrate the NT scATAC‐seq and HT scATAC‐seq dataset. The weighted nearest neighbor (WNN) procedure^[^ ^83^ ^]^ implemented in Seurat (v.4.1.1) was used to integrate scRNA‐seq and scATAC‐seq that were collected in the same cells.

### TF Regulatory Network Analysis and Single‐Nucleus Consensus Weighted Gene Co‐Expression Network Analysis

The pseudotime of each tapetal cell assigned by Monocle2 (v.2.22.0) was normalized from 0 to 1. Gene regulatory network inference was calculated on dynamic TFs using SCODE with parameter z setting as 4, replicate setting as 50.^[^ ^61^ ^]^ A single‐nucleus consensus weighted gene coexpression network analysis (scWGCNA) was used within cells that disappeared under HT in Figure 6F by hdWGCNA R package (v.0.2.01).^[^ ^91^ ^]^ The resulting adjacency matrix was then transformed into a topological overlap matrix. The modules were defined using specific module‐cutting parameters, including a minimum module size of 100 genes, a deepSplit score of 4, and a correlation threshold of 0.2. Modules with a correlation greater than 0.8 were merged. These central genes were visualized with intra‐modular connectivity (kME) values >0.2.

### In Situ RNA Hybridization

Briefly, a 150–200 bp fragment was amplified from the H05 cDNA. The PCR product was cloned into the pGEM‐T‐Easy vector (Promega, USA) and sequenced. Sense and antisense probes were transcribed in vitro from the T7 or SP6 promoter with respective RNA polymerases using the digoxigenin RNA labelling kit (Roche, Germany). Tissue sections were fixed in 50% FAA [10% formalin, 5% acetic acid, and 50% ethanol (v/v) in RNase‐free water]. After dehydration and embedding of the tissue in paraffin wax, the sample blocks were sectioned into 8‐µm slices using the microm HM 340E microtome (Thermo Scientific, USA) and were applied to RNase‐free glass slides (Solarbio, China). Hybridization was detected by using the antidigoxigenin‐alkaline phosphatase conjugate (Roche, dilution 1:1000), visualized by incubation with NBT/BCIP stock solution (Roche, dilution 1:50), and images were captured using a Zeiss Axioscope A1 microscope (Carl Zeiss, Germany). Primers are listed in Table S8 (Supporting Information).

### Immunofluorescence

To determine the accumulation of pectic polysaccharides in anthers of H05 plants under NT and HT conditions, JIM5 and JIM7 antibodies (Agrisera, Vannas, Sweden) were used to detect de‐esterified homogalacturonan and esterified homogalacturonan, respectively. The anthers section of H05 under NT and HT during stage 7–12 used in this assay was same as in situ hybridization. The following processes were performed according to Wu et al. (2022).^[^ ^64^ ^]^ Fluorescence signals were observed using a confocal microscope (Olympus FV1200).

### Luciferase Assay

For detecting the temperature‐dependent protein abundance, *pGh* *CYP703A2* and *pΔGh* *CYP703A2* were cloned into vector pGreenII 0800‐LUC using primers 0800‐CYPpro‐F and 0800‐CYPpro‐R. 35S:MS188 were cloned into vector pGreenII 62‐SK using primers 62SK‐MS188‐F and 62SK‐MS188‐R. All the constructs were introduced into *A. tumefaciens* strain *GV3101*. The infiltration buffer was made of 10 mm MES, 1 mm MgCl_2_ and 0.2 mm Acetosyringone. After infiltration, all plants were incubated at 28 °C for 24 h, and plant subsequently transferred to incubation at 28 °C for 24 h as NT samples and to incubation at 36 °C for 24 h as HT samples. Primers are listed in Table S8 (Supporting Information).

### Availability of Data and Materials

All the raw sequencing data generated during the current study are available in the NCBI BioProject database under accession number PRJNA1010459. Other relevant materials are available from the corresponding authors on reasonable request.

## Conflict of Interest

The authors declare no conflict of interest.

## Author Contributions

Y.L.L. and H.M. contributed equally to this project (co‐first authors). L.M. and X.Z. are both co‐last authors. Y.L.L. performed bioinformatic analysis and wrote the main manuscript text, H.M. performed the major genetic and biochemical analyses, Y.W., Y.M., J.K. supervised the research; YW.L., J.Y., R.Z., D.Y. performed anther sampling; L.M., X.Z., and K.L. revised the manuscript. All authors reviewed the manuscript.

## Supporting information

Supporting Information

Supplemental Table 1

Supplemental Table 2

Supplemental Table 3

Supplemental Table 4

Supplemental Table 5

Supplemental Table 6

Supplemental Table 7

Supplemental Table 8

Supplemental Movie 1

## Data Availability

The data that support the findings of this study are available from the corresponding author upon reasonable request.
