# Supplementary material for: Single‐Cell Transcriptome Atlas and Regulatory Dynamics in Developing Cotton Anthers
Source: Adv Sci (Weinh). 2023 Nov 17;11(3):2304017. doi: 10.1002/advs.202304017 (PMC10797427; doi:10.1002/advs.202304017)
Supplement: Supplementary file 1 — Supporting Information [file ADVS-11-2304017-s008.pdf]

ADVANCED SCIENCE
Open Access

Supporting Information

for *Adv. Sci.*, DOI 10.1002/advs.202304017

Single-Cell Transcriptome Atlas and Regulatory Dynamics in Developing Cotton Anthers

Yanlong Li, Huanhuan Ma, Yuanlong Wu, Yizan Ma, Jing Yang, Yawei Li, Dandan Yue, Rui Zhang, Jie Kong, Keith Lindsey, Xianlong Zhang and Ling Min**

Supporting Information

Single-Cell Transcriptome Atlas and Regulatory Dynamics in Developing Anthers

Yanlong Li, Huanhuan Ma, Yuanlong Wu, Yizan Ma, Jing Yang, Yawei Li, Dandan Yue, Rui Zhang, Jie Kong, Keith Lindsey, Xianlong Zhang, Ling Min**

Dr.YL.Li, Dr.H.Ma, Dr.Y.Wu, Dr.Y.Ma, Dr.J.Yang, Dr.YW.Li, Dr.D.Yue, Dr.R.Zhang, Prof.X.Zhang, Prof.L.Min.
National Key Laboratory of Crop Genetic Improvement & Hubei Hongshan Laboratory, Huazhong Agricultural University
Wuhan 430070, Hubei, China
e-mail: lingmin@mail.hzau.edu.cn and xlzhang@mail.hzau.edu.cn

Prof.K.Lindsey
Department of Biosciences
Durham University
Durham, UK

Prof.J.Kong
Institute of Economic Crops
Xinjiang Academy of Agricultural Sciences
Xinjiang 830091, China

Keywords: single-cell RNA sequencing (scRNA-seq), single cell multi-omics, anther, high temperature, tapetum

Supplementary Figures

Supplementary Figure 1

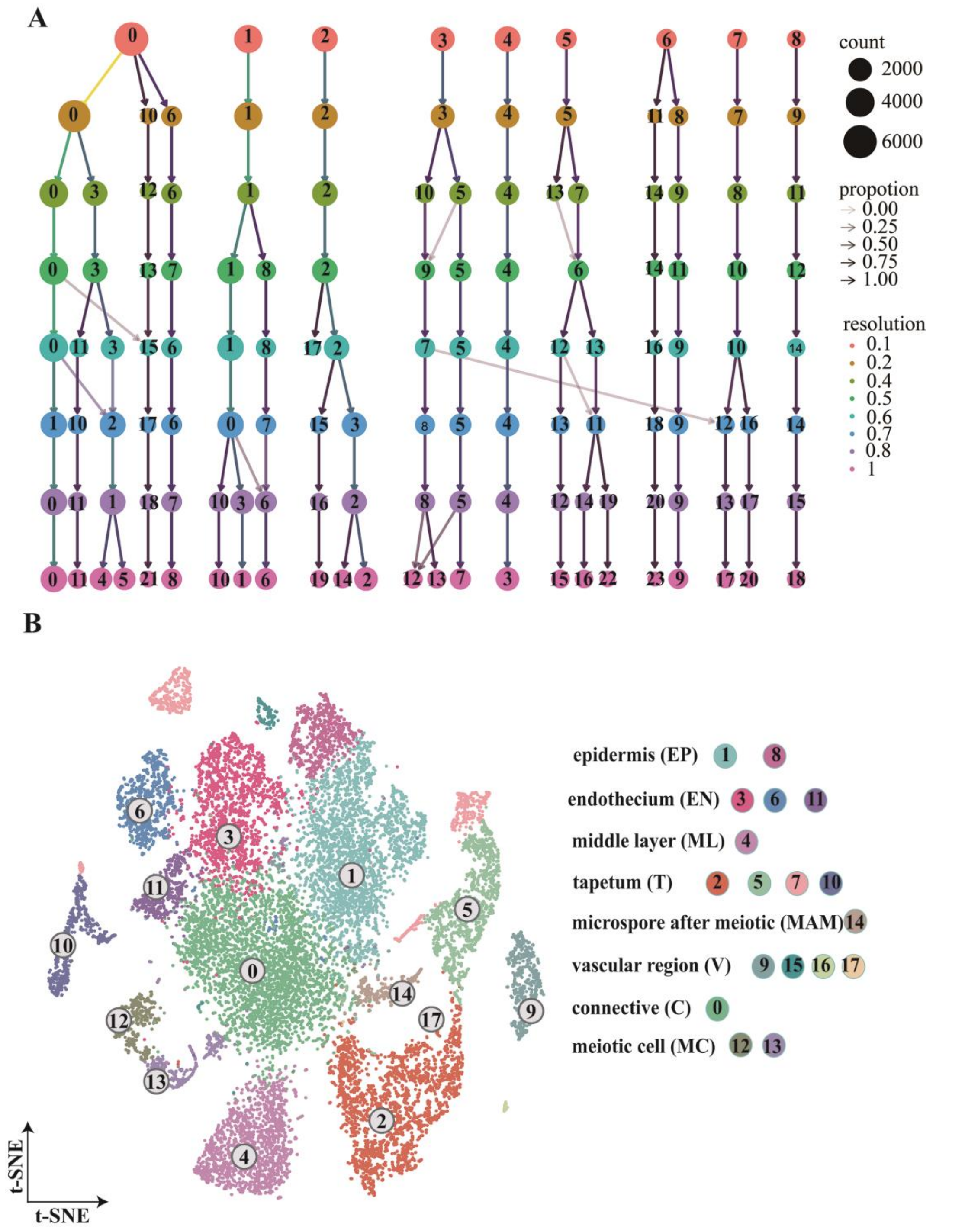

Supplementary Figure 1. Cell clustering of cotton anthers visualized by *t*-SNE. (A) Clutree showing clustering under different resolutions set in Seurat. Circle size, cell number; circle color, resolution; arrow transparency, proportion in the cluster. (B) *t*-SNE visualization of 18 cell clusters of cotton anthers. Each dot denotes a single cell. Colors denote corresponding cell clusters as in Figure 1C.

Supplementary Figure 2

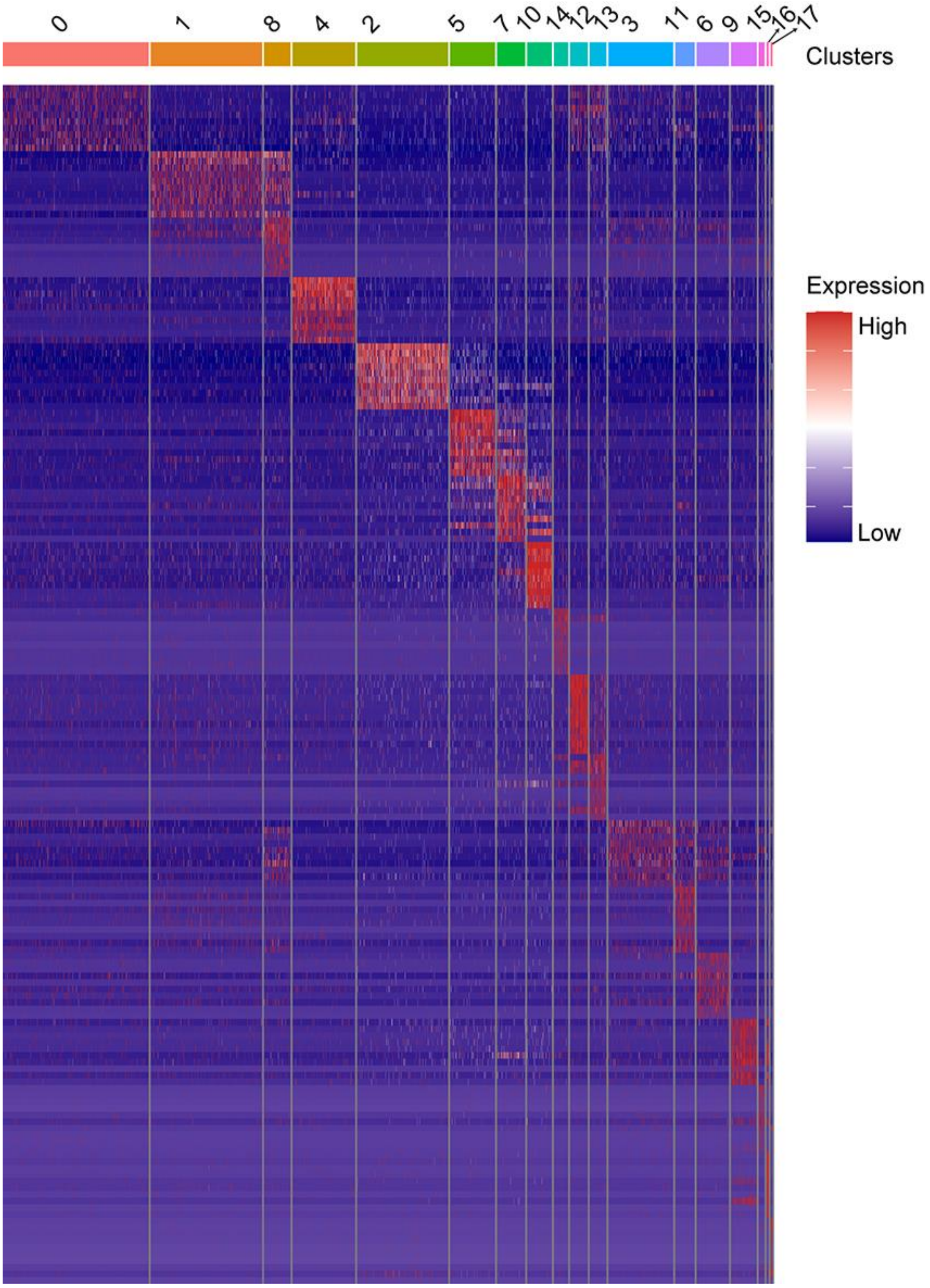

Supplementary Figure 2. Heatmap of the top 10 marker genes from each cell cluster in cotton anthers. Each square represents the average expression level per cell; number represent cell cluster under the resolution of 0.6.

Supplementary Figure 3

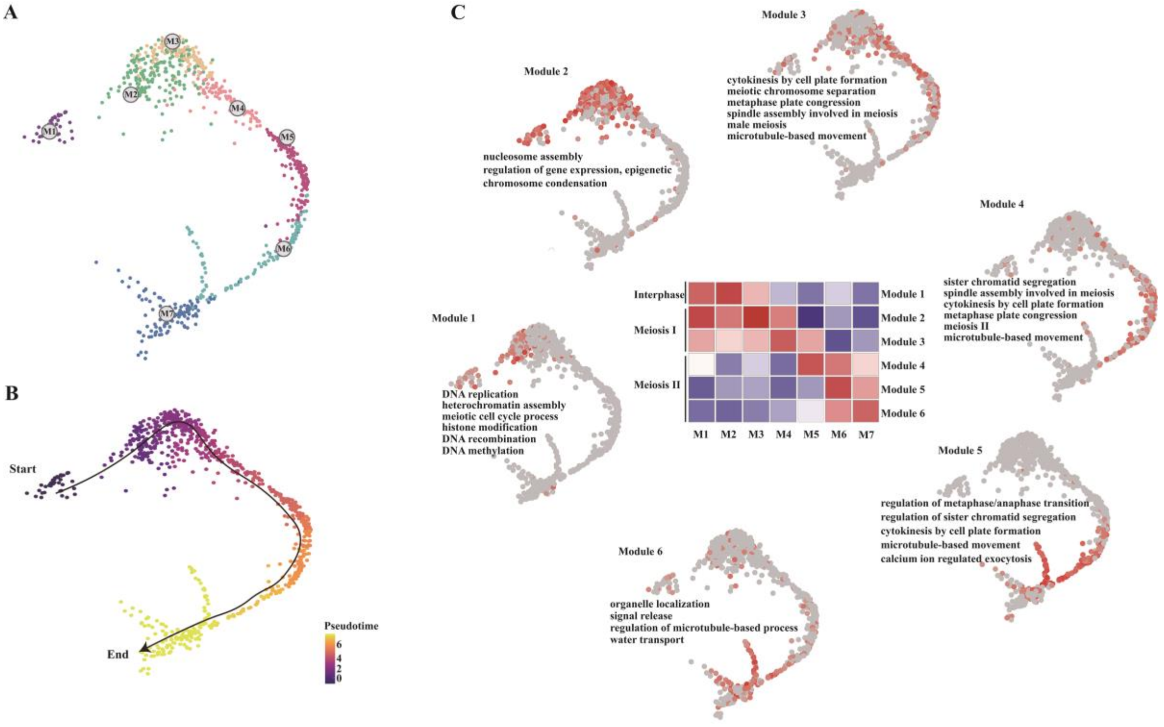

Supplementary Figure 3. Developmental trajectory of meiotic cells. (A) UMAP projections showing meiotic cell populations. M1 to M7, sub-cell clusters. (B) UMAP of the pseudotime trajectory of meiotic cells by using Monocle3. (C) Modules of co-regulated differentially expressed genes and functional gene set enrichment pathways of each module.

Supplementary Figure 4

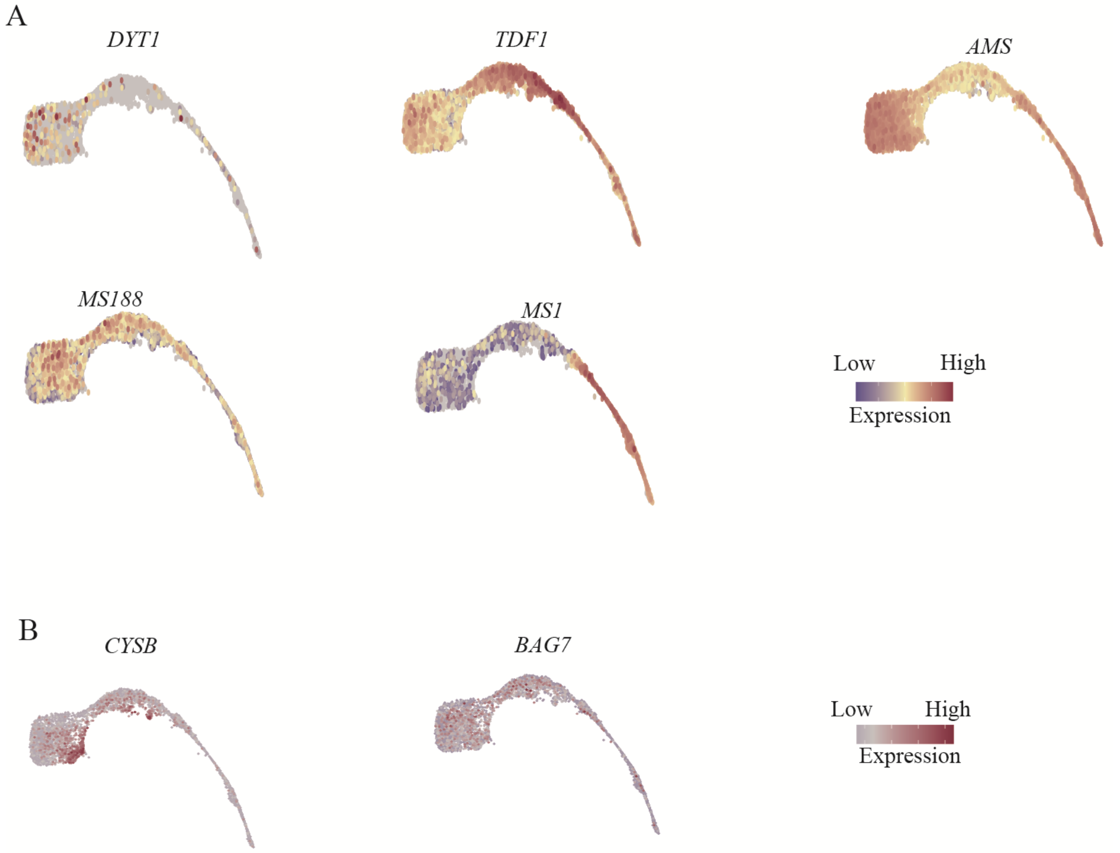

Supplementary Figure 4. UMAP plots showing the expression pattern of the special genes of tapetum. (A). Expression pattern of a genetic pathway involving DYT1-TDF1-AMS- MS188-MS1 in tapetum development. (B) UMAP plots of CYSB and BAG7.

Supplementary Figure 5

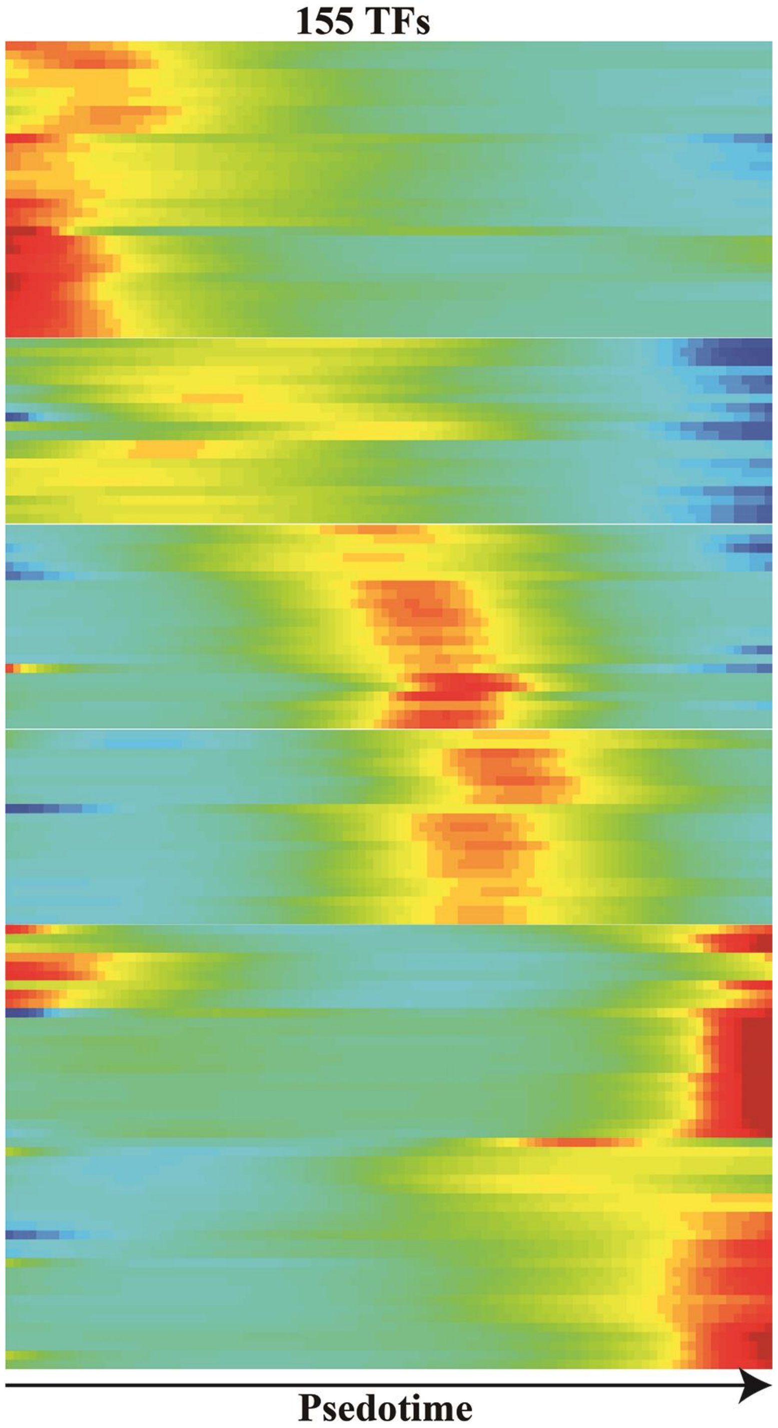

Supplementary Figure 5. Expression heatmap of 155 transcription factors (TFs) in tapetal cells cluster along pseudotime.

Supplementary Figure 6

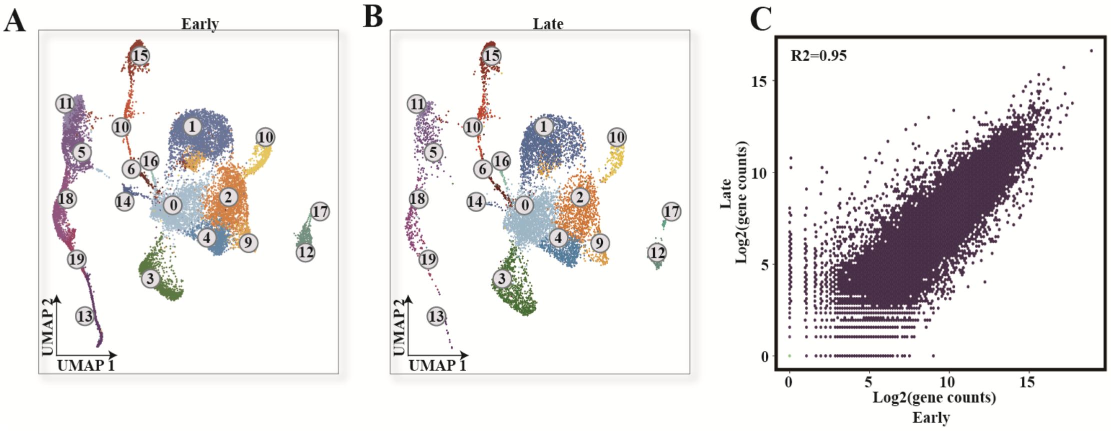

Supplementary Figure 6. Comparison of two NT samples. (A-B) UMAP plots of earlier NT sample (A) and later NT sample (B) after integration using Seurat. Labels denote corresponding cell clusters as in **Figure. 5A**. (C) The expression correlation scatter map of earlier NT sample and later NT sample. Correlations were calculated using counts for each gene. R^2 represents the pearson correlation coefficient.

Supplementary Figure 7

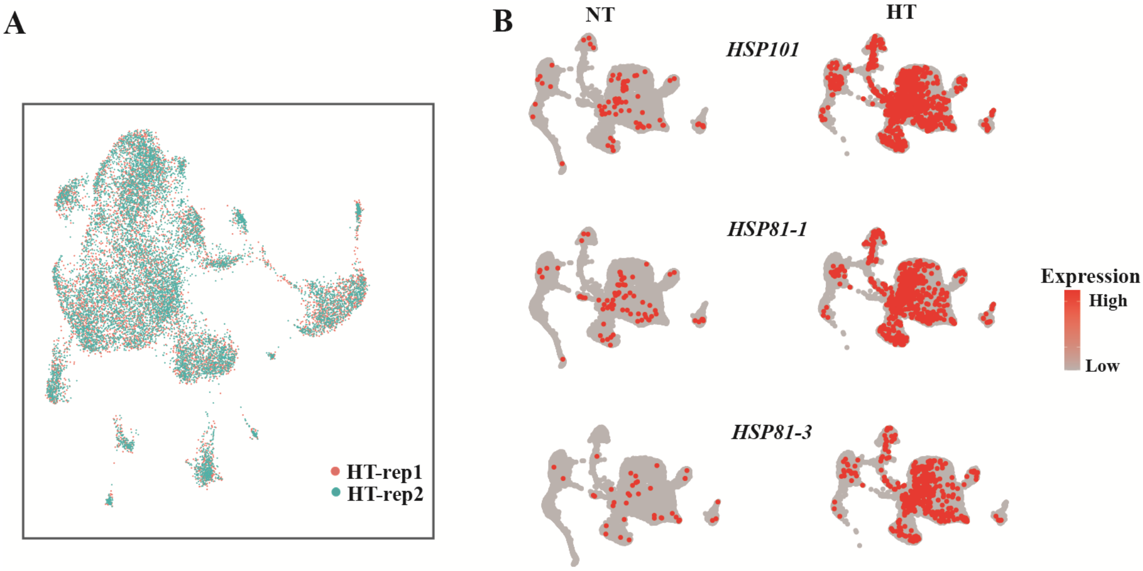

Supplementary Figure 7. UMAP plots showing the expression pattern of *HSPs*. (A) UMAP visualization of two biological replicates of cotton anthers under high temperature. (B) UMAP plot showing the expression pattern of *HSP101*, *HSP81-1* and *HSP81-3* under normal temperature and high temperature.

Supplementary Figure 8

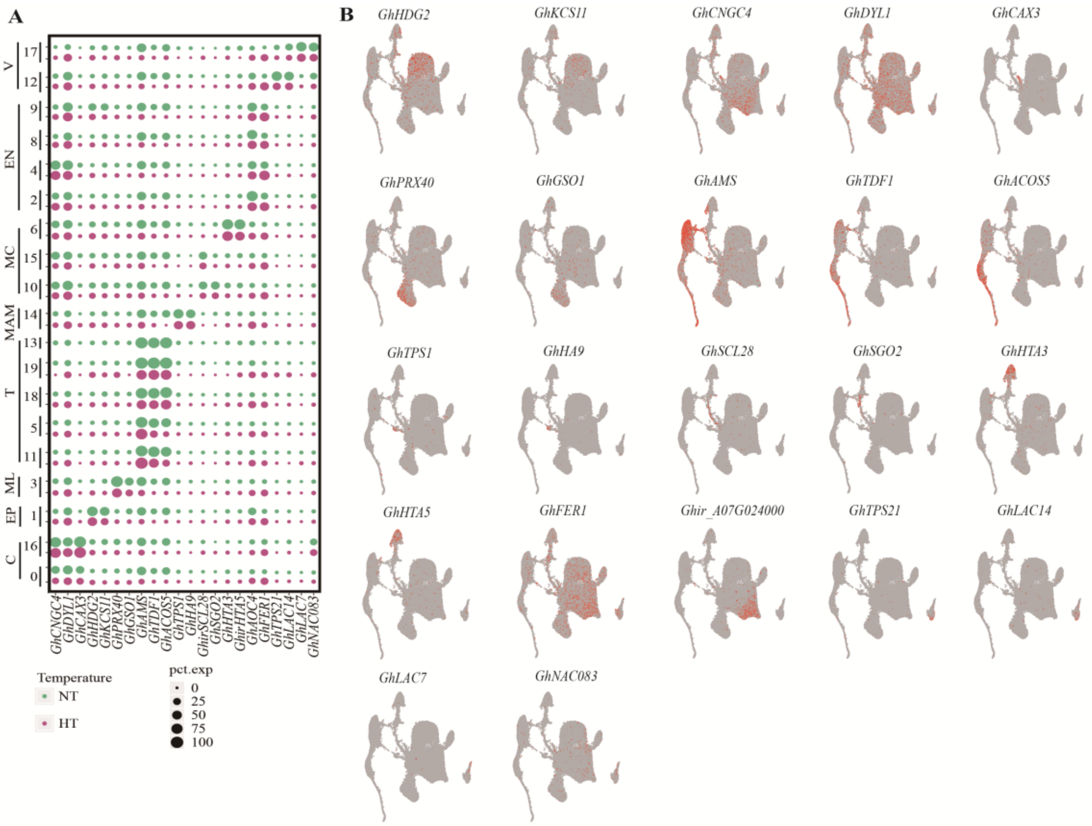

Supplementary Figure 8. Conservative genes under NT and HT. (A) Expression pattern of representative cluster-conserved marker genes. Dot diameter, proportion of cluster cells expressing a given gene. (B) UMAP plot showing the selected cluster-conserved marker genes for different cell types. EP, epidermis; EN, endothecium; ML, middle layer; T, tapetum; MAM, microspore after meiotic; V, vascular region; C, connective; MC, meiotic cell.

Supplementary Figure 9

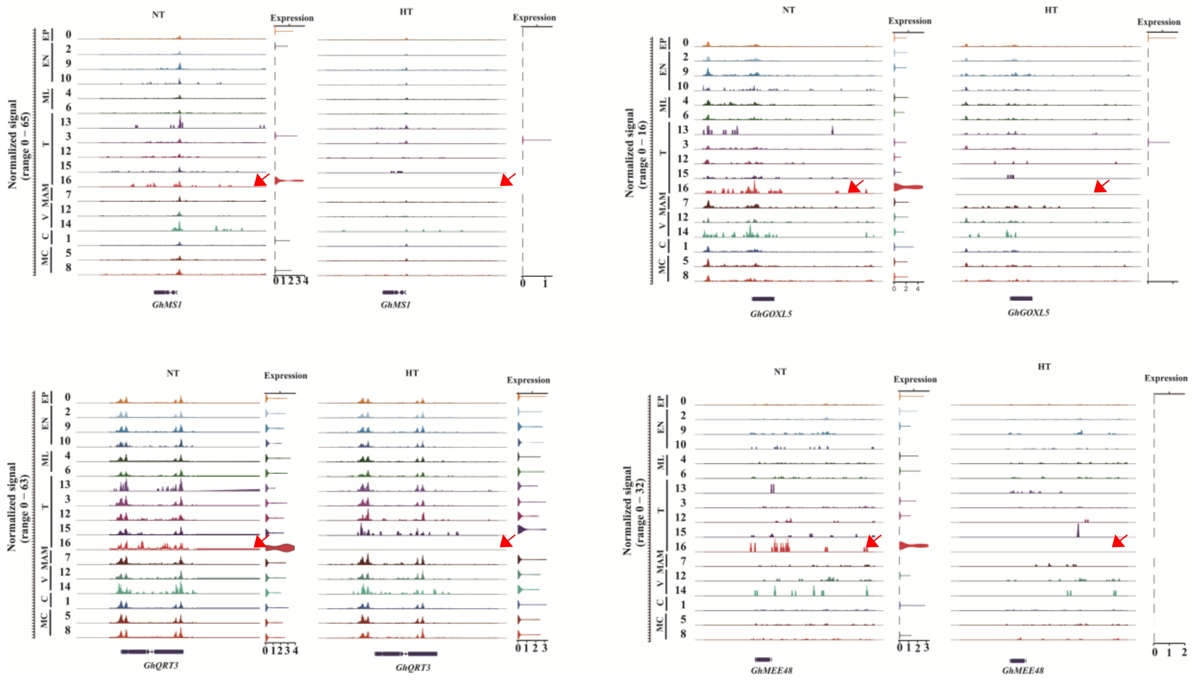

–

Supplementary Figure 9. Visualization of chromatin accessibility tracks of the gene locus associated with pollen wall formation across all clusters. The red arrows represent the tapetal cells responsible for pollen wall synthesis. EP, epidermis; EN, endothecium; ML, middle layer; T, tapetum; MAM, microspore after meiotic; V, vascular region; C, connective; MC, meiotic cell. NT, normal temperature; HT, high temperature.

Supplementary Figure 10

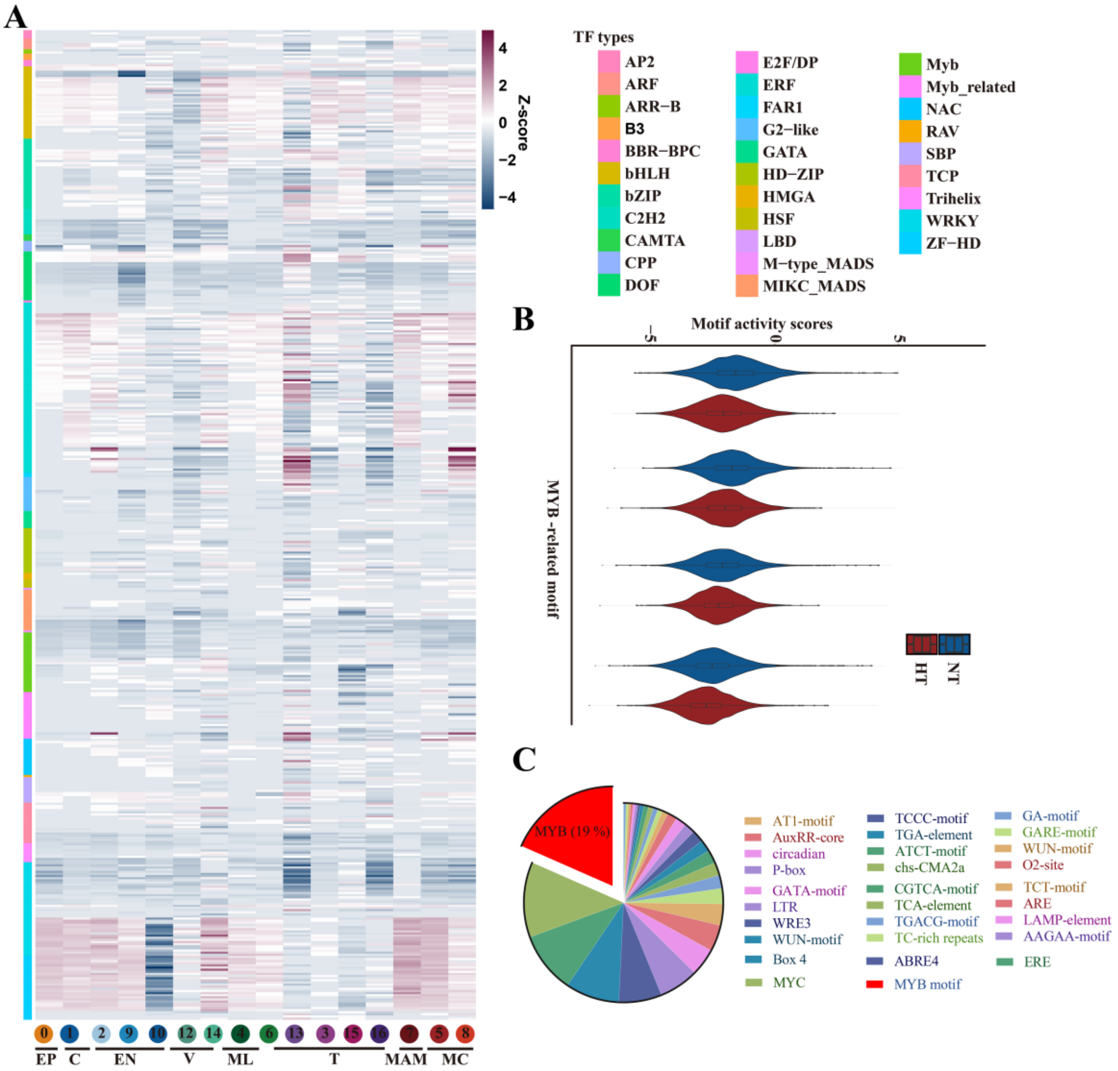

Supplementary Figure 10. Changes in chromatin accessibility of 465 TF motifs under HT. (A) The 465 TF motif enrichment differences (the log2 fold change) across all clusters under NT and HT scale by row with z-score. (B) chromVAR motif activity scores for the MYB-related motifs under NT and HT. (C) Statistics on the types of transcription factor motif on the promoters of pollen wall synthesis-related genes. EP, epidermis; EN, endothecium; ML, middle layer; T, tapetum; MAM, microspore after meiotic; V, vascular region; C, connective; MC, meiotic cell. NT, normal temperature; HT, high temperature.

Supplementary Figure 11

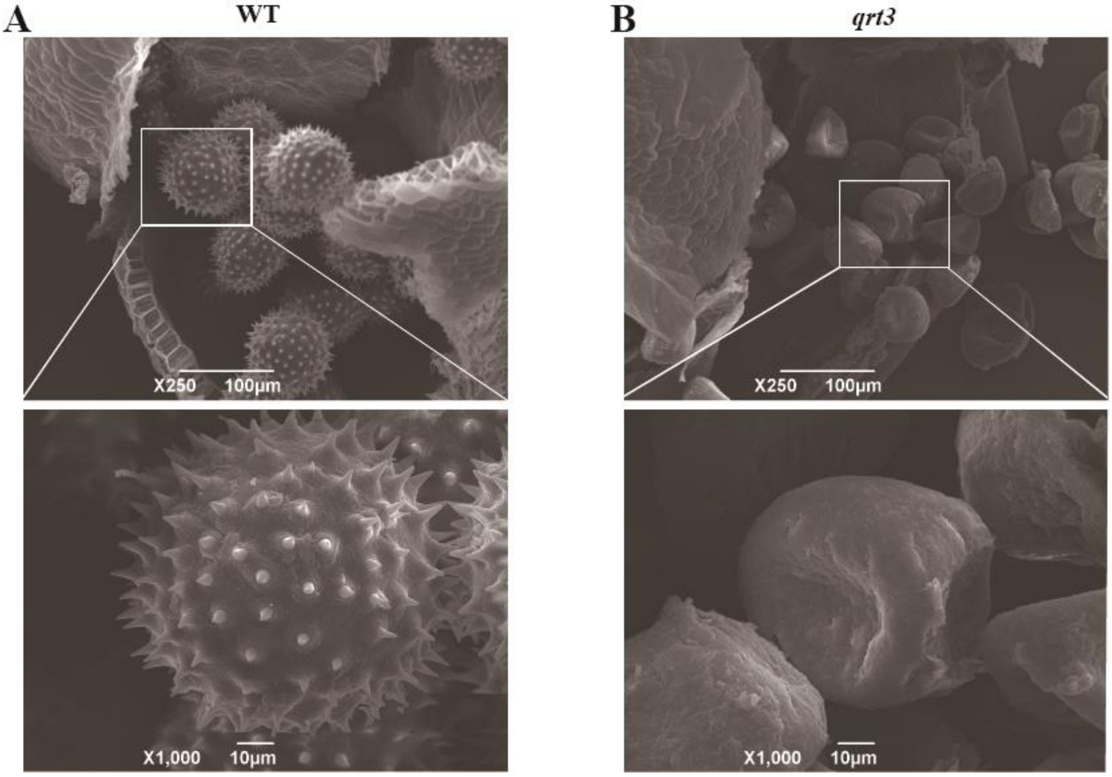

Supplementary Figure 11. The phenotype of pollen of *qrt3*. (A-B) Scanning electron microscopy analysis of pollen grains of the WT (A) and *qrt3* (B) at stage 12.

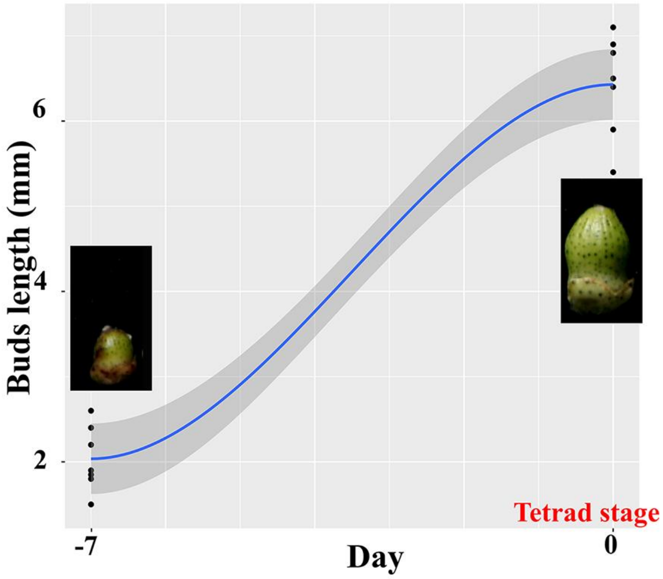

Supplementary Figure 12. Growth curve of cotton buds at tetrads stage under high temperature
